# Targeting mitochondrial dynamics of morphine-responsive dopaminergic neurons ameliorates opiate withdrawal

**DOI:** 10.1172/JCI171995

**Published:** 2024-01-18

**Authors:** Changyou Jiang, Han Huang, Xiao Yang, Qiumin Le, Xing Liu, Lan Ma, Feifei Wang

**Affiliations:** 1 State Key Laboratory of Medical Neurobiology and MOE Frontiers Center for Brain Science, Institutes of Brain Science and School of Basic Medical Sciences, Departments of Neurosurgery and Hand Surgery, Huashan Hospital, Fudan University, Shanghai, China.; 2 Research Unit of Addiction Memory, Chinese Academy of Medical Sciences (2021RU009), Shanghai, China.

**Keywords:** Neuroscience, Therapeutics, Addiction, Mitochondria, Neurological disorders

## Abstract

Converging studies demonstrate the dysfunction of the dopaminergic neurons following chronic opioid administration. However, the therapeutic strategies targeting opioid-responsive dopaminergic ensembles that contribute to the development of opioid withdrawal remain to be elucidated. Here, we used the neuronal activity-dependent Tet-Off system to label dopaminergic ensembles in response to initial morphine exposure (Mor-Ens) in the ventral tegmental area (VTA). Fiber optic photometry recording and transcriptome analysis revealed downregulated spontaneous activity and dysregulated mitochondrial respiratory, ultrastructure, and oxidoreductase signal pathways after chronic morphine administration in these dopaminergic ensembles. Mitochondrial fragmentation and the decreased mitochondrial fusion gene mitofusin 1 (*Mfn1*) were found in these ensembles after prolonged opioid withdrawal. Restoration of *Mfn1* in the dopaminergic Mor-Ens attenuated excessive oxidative stress and the development of opioid withdrawal. Administration of Mdivi-1, a mitochondrial fission inhibitor, ameliorated the mitochondrial fragmentation and maladaptation of the neuronal plasticity in these Mor-Ens, accompanied by attenuated development of opioid withdrawal after chronic morphine administration, without affecting the analgesic effect of morphine. These findings highlighted the plastic architecture of mitochondria as a potential therapeutic target for opioid analgesic-induced substance use disorders.

## Abstract 2

Dopaminergic ensemble-specific mechanisms in the ventral tegmental area underly opiate withdrawal and targeting mitochondrial dynamics may provide a strategy to alleviate withdrawal.

## Graphical abstract

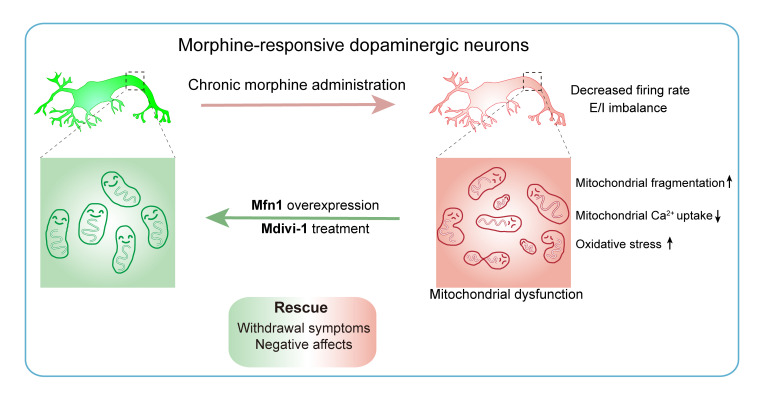

## Introduction

Prescription opioids, such as morphine, are the most effective clinical analgesics. However, their clinical utility is limited by analgesic tolerance and reinforcing effects (1, 2). Opioids exert potent euphoric effects during the binge/intoxication phase and induce negative reinforcement and aversive effects during withdrawal (3). The ventral tegmental area (VTA), a heterogeneous midbrain structure containing dense distribution of opioid receptors and endogenous opioid peptides, is thought be a critical site for opioid-induced reinforcement and drug seeking (1, 4–6). Emerging evidence suggests that dopaminergic dysfunction in the VTA underlies the pathophysiology of several psychiatric disorders, including schizophrenia, addiction, and depression (7–10). Systemic or VTA injection of morphine increases the firing rate of dopaminergic neurons (11). Chronic exposure and withdrawal from morphine alter the morphology and plasticity of dopaminergic neurons in the VTA (12, 13).

Sparse neuronal populations recruited by addictive drugs might be involved in the encoding and expression of drug-mediated associations (14–16). The advancement of techniques for labeling and manipulating immediate early gene–expressing (IEG-expressing) cells activated by either drugs or drug-associated stimuli has been used to causally establish their involvement in drug responses (17). Our recent study shows that the inhibitory transmission to VTA ensembles labeled by an initial morphine injection (morphine ensembles, Mor-Ens) is enhanced following chronic morphine administration and mediates the negative affects during opiate withdrawal (18). However, the hub signaling pathways and molecular changes of the opioid-responsive ensembles after chronic opioid administration remain to be elucidated.

Transcriptional responses are elicited in the brain by a variety of stimuli, and have been implicated in many biological processes and diseases (19–23). Gene expression profiling facilitates unbiased discovery to identify the molecules or pathways in specific functional pathways involved in physiological or pathological changes (24, 25). Transcriptional responses triggered by the addictive drug stimulus may shape neuronal circuits to induce addictive behavior and have been implicated in leading to long-lasting cellular adaptations (15, 17, 26, 27). The transcriptomic dynamics of the VTA dopaminergic Mor-Ens after chronic opiate administration are unclear, and the molecular basis that mediates the dysfunction of these ensembles involved in behavioral alterations during opioid withdrawal needs to be determined.

In this study, transcriptomic analysis of VTA dopaminergic Mor-Ens reveals that chronic morphine administration alters the genes associated with mitochondrial function and oxidative stress pathways. Mitochondria-generated ATP is required for the establishment of appropriate electrochemical gradients and reliable synaptic transmission in neurons. Distinct cellular events that induce oxidative stress or disrupt mitochondrial homeostasis trigger neuropathology (28, 29). The highly dynamic mitochondrial structure and functional network of neurons play critical roles in maintaining energy homeostasis in response to various stimuli (28). Dopaminergic neurons have high energy demands, making them particularly sensitive to mitochondrial dysfunction (30). The interplay between mitochondrial defects and abnormalities in dopamine metabolism have been implicated in addiction, attention deficit/hyperactivity disorder, and schizophrenia (31). In vivo results of this study show that chronic morphine administration causes excessive mitochondrial fragmentation and oxidative stress, as well as the impaired firing and E/I ratio in dopaminergic Mor-Ens. Remarkably, targeting the mitochondrial dynamics with overexpressing the mitochondrial fusion gene Mfn1 or administrating Mdivi-1, a mitochondrial fission inhibitor, attenuates morphine-induced cellular maladaptation in the VTA dopaminergic Mor-Ens and alleviates the development of withdrawal symptoms and negative affects of morphine. This study provides potential therapeutic strategies targeting the plastic architecture of mitochondria to prevent the side effects of opioid analgesics.

## Results

### Decreased activity of dopaminergic morphine-responsive neurons in the VTA mediates conditioned aversion and anxiety during morphine withdrawal.

**Figure 1:**
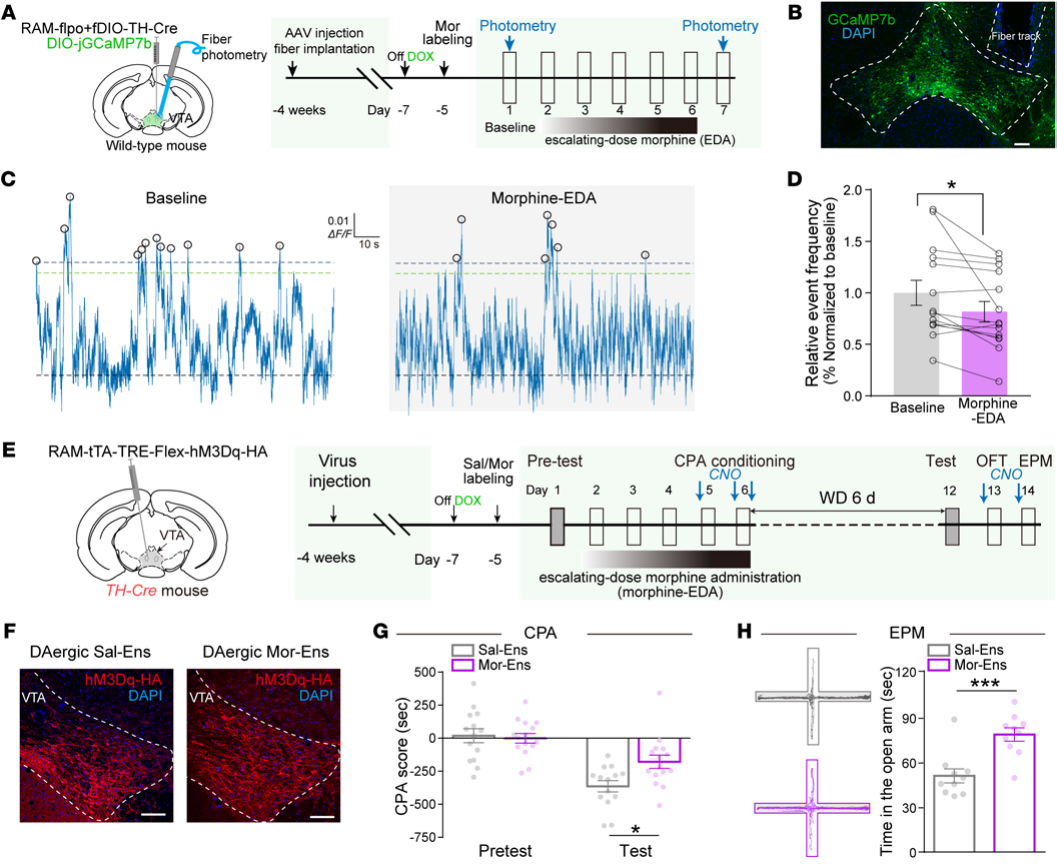
Dysregulation of the spontaneous activity in the VTA dopaminergic Mor-Ens mediates withdrawal-induced aversion and anxiety after chronic morphine administration. (**A**) Schematic of virus injection and fiber photometry recordings. Viruses combining Cre-loxp and Flpo-FRT systems were used to label TH^+^ neuronal ensembles with GCaMP7b, and the optic fiber was unilaterally implanted in the VTA of WT mice. (**B**) Representative images of GCaMP7b expression in the VTA. Dashed white lines outline the VTA and optic fiber tract. Green, GCaMP7b; Blue, DAPI. Scale bar: 200 μm. (**C**) Representative photometric traces of GCaMP7b signals in Mor-Ens before and after morphine EDA. Marked circles indicate detected events above the threshold. (**D**) Relative frequency of calcium events in Mor-Ens. Paired *t* test, *n* = 14. (**E**) Experimental scheme of the ensembles labeling and behavioral testing in *TH-Cre* mice. *AAV-RAM-tTA-TRE-hM3Dq-HA* was injected into the VTA of *TH-Cre* mice to label RAM-driven expression of hM3Dq-HA in TH^+^ neuronal ensembles. (**F**) Representative images of hM3Dq-HA expression in VTA ensembles. Red, hM3Dq-HA; Blue, DAPI. Scale bar: 100 μm. White dashed line outlines the boundary of VTA. (**G** and **H**) The effects of CNO activation of Mor-Ens on CPA and anxiety during morphine withdrawal. CPA score (**G**), the representative traces in EPM test, and the quantification of time in the open arm (**H**) are represented. 2-way RM ANOVA, Sal-Ens, *n* = 14; Mor-Ens, *n* = 15 in **G**. Unpaired *t* test, *n* = 10 mice per group in **H**. Data are presented as mean ± SEM; *P < 0.05, ***P < 0.0001.

Plasticity of neurons in the VTA is critical for the development of morphine withdrawal. Neuronal ensembles responding to initial morphine exposure (Mor-Ens) in the VTA preferentially project to the NAc and induce dopamine-dependent positive reinforcement (18). To investigate the maladaptive changes in the dopaminergic Mor-Ens, the Cre-Loxp and Flpo-Frt dependent robust activity marking (RAM) system was used to label jGCaMP7b in these dopaminergic Mor-Ens in the absence of doxycycline (Dox) (Figure 1A). *AAV-RAM-TTA-TRE-flpo*, *AAV-fDIO-TH-Cre*, and *AAV-DIO-jGCaMP7b* viruses were delivered, and the optic fibers were unilaterally implanted in the VTA. The frequency of spontaneous Ca^2+^ events of these ensembles were recorded before and after escalating dose administration of morphine (morphine EDA) in the home cage (Figure 1, A and B). The frequency of Ca^2+^ events decreased after morphine EDA (day 7) compared with baseline (day 1) (Figure 1, C and D), but did not change by saline treatments (Supplemental Figure 1, A–C; supplemental material available online with this article; https://doi.org/10.1172/JCI171995DS1). Immunostaining showed that the majority of the GCaMP7b^+^ ensembles were restricted in the VTA and colabeled with tyrosine hydroxylase (TH) (Supplemental Figure 1, D and E). These data suggest a decrease in spontaneous neuronal activity of VTA dopaminergic Mor-Ens after morphine EDA.

To evaluate the role of dopaminergic Mor-Ens in negative affects during morphine withdrawal, *AAV-RAM-TTA-TRE-Flex-hM3Dq(Gq)-HA* virus was delivered into the VTA of *TH-Cre* transgenic mice. In the absence of Dox, hM3Dq was labeled in dopaminergic Mor-Ens or saline ensembles (Sal-Ens) (Figure 1, E and F). The hM3Dq-labeled ensembles were restricted in the VTA and colocalized with TH (Supplemental Figure 2, A and B). Clozapine-N-oxide (CNO, 2 mg/kg) was injected 30 minutes before each aversion conditioning session, as well as the open field and elevated plus maze (EPM) tests (Figure 1E). Chemogenetic-activated dopaminergic Mor-Ens of the mice reduced the conditioned place aversion (CPA) score and increased the time spent in the open arms of EPM (Figure 1, G and H), while having no effect on locomotor activity (Supplemental Figure 2, C and D). These data suggest that the restoration of dopaminergic Mor-Ens activities by designer receptors exclusively activated by designer drugs (DREADDs) during morphine withdrawal alleviates aversion and anxiety in mice.

### Chronic morphine administration dysregulates the signal pathways of mitochondrial functions in the VTA dopaminergic Mor-Ens.

**Figure 2:**
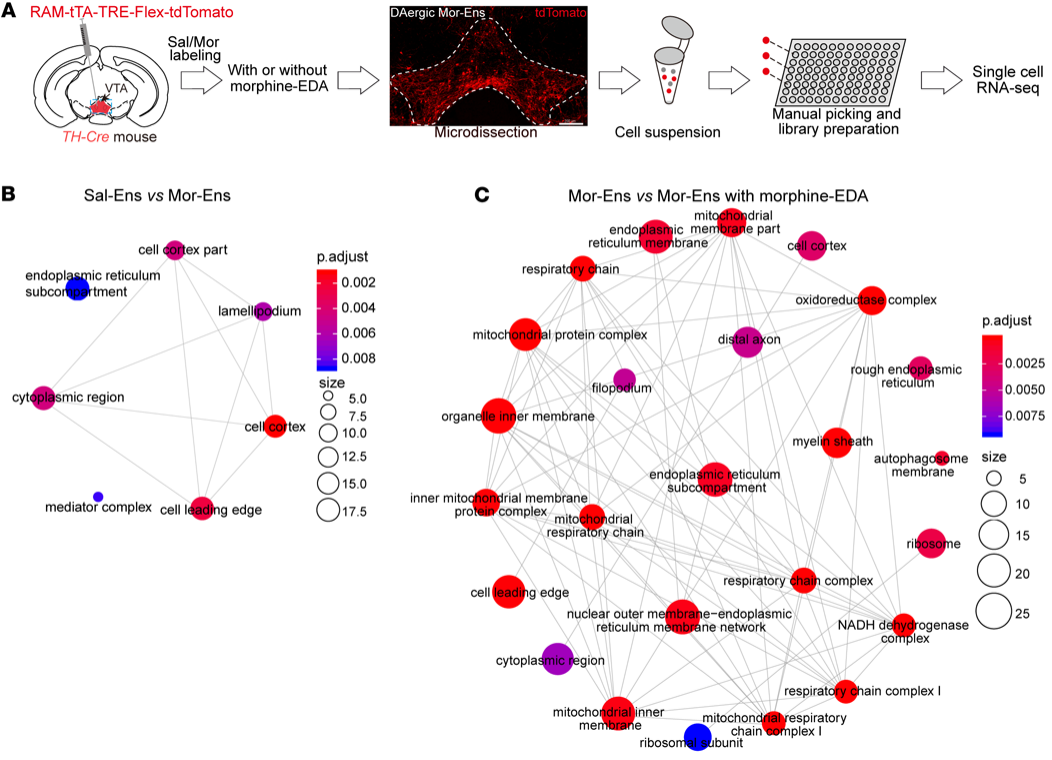
Chronic morphine administration alters dysregulation of the mitochondria-related signaling pathways in the VTA dopaminergic Mor-Ens. (**A**) Experimental scheme for single-cell RNA–Seq of the VTA dopaminergic ensembles. Red, tdTomato. Scale bar: 200 μm. (**B**) Signaling network enrichment analysis between dopaminergic Sal-Ens and Mor-Ens. (**C**) Signaling network enrichment analysis between dopaminergic Mor-Ens treated without or with morphine EDA groups. *n* = 4 mice per group.

To characterize cell type–specific adaptations by initial and chronic morphine exposure in the VTA dopaminergic neurons, *AAV-RAM-TTA-TRE-Flex-tdTomato* virus was injected into the VTA of *TH-Cre* transgenic mice. The dopaminergic Mor/Sal-Ens (tdTomato^+^ cells) from the dissected VTA were manually picked (approximately 1 % of viable cells). Single-cell RNA-Seq was used to determine the transcriptional differences of Sal-Ens, Mor-Ens, and Mor-Ens with morphine EDA groups (Sal-Ens group, 58 cells; Mor-Ens group, 54 cells; Mor-Ens with morphine EDA group, 56 cells; Figure 2A and Supplemental Figure 2, E and F). A total of 325 differentially expressed genes (DEGs) were identified between Sal-Ens and Mor-Ens, while 450 genes were altered by Morphine EDA in Mor-Ens (Supplemental Figure 3A). Over-representation analysis revealed enrichment of genes in protein kinase A binding and ubiquitin protein ligase activity between Sal-Ens and Mor-Ens, while pathways including nadh dehydrogenase activity, oxidoreductase activity, and phosphoprotein phosphatase activity were changed in Mor-Ens by Morphine EDA (Supplemental Figure 3, B and C).

Network enrichment analysis indicates that dopaminergic Sal-Ens and Mor-Ens exhibited significant differences in endoplasmic reticulum, lamellipodium, cell cortex, and cytoplasmic region–related pathways (Figure 2B), whereas DEGs in dopaminergic Mor-Ens changed by Morphine EDA were enriched in the pathways including mitochondrial respiratory chain, mitochondrial protein complex, mitochondrial ultrastructure, nadh dehydrogenase complex, oxidoreductase complex, and autophagy (Figure 2C), suggesting that chronic morphine administration may lead to dysregulated mitochondrial function in dopaminergic Mor-Ens, which is fundamental for metabolic homeostasis (32).

### Chronic morphine administration impairs mitochondrial Ca^2+^ transport and promotes mitochondrial fragmentation in dopaminergic Mor-Ens, accompanied by impaired mitochondrial respiration and increased mitophagy in the VTA.

**Figure 3:**
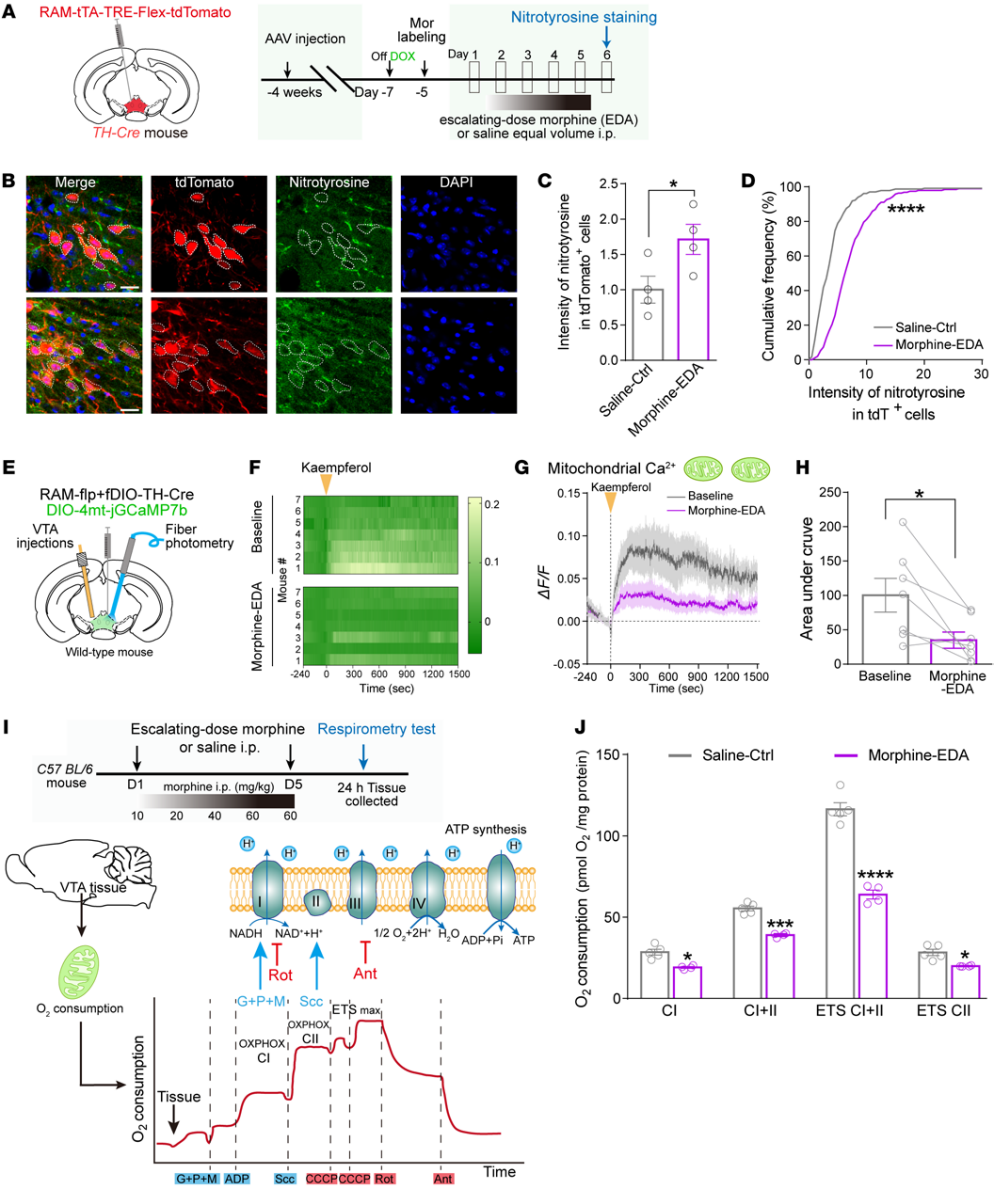
Chronic morphine administration induces increased oxidative stress and impairs Ca^2+^ transport in dopaminergic Mor-Ens and mitochondrial respiration in the VTA. (**A**) Experimental scheme to assess the oxidative stress in the dopaminergic Mor-Ens of VTA from mice with or without morphine EDA. (**B**) Representative images of nitrotyrosine staining of the brain slices containing VTA. White dashed lines outline Mor-Ens. Red, tdTomato; Green, nitrotyrosine; Blue, DAPI. Scale bar: 20 μm. (**C**) The normalized expression level of nitrotyrosine in VTA tdTomato^+^ ensembles in saline and morphine EDA groups. Unpaired *t* test, *n* = 4 mice per group. (**D**) Cumulative frequency distribution of nitrotyrosine intensity in tdTomato^+^ neurons. 2-sample Kolmogorov-Smirnov test, Saline Ctrl, 229 cells from 4 mice; Morphine EDA, 194 cells from 4 mice. (**E**) Schematic of fiber photometry setup for detecting mitochondrial Ca^2+^ signal in Mor-Ens in freely moving mice. (**F**) Heatmap of relative mito-GCaMP fluorescence intensity in Mor-Ens after intracerebral injection of kaempferol into VTA (1.6 μL, 2 nmol/μL) in mice with or without morphine EDA. (**G**) Average ΔF/F (%) and (**H**) the AUC quantification of mito-GCaMP fluorescence. Dashed vertical line indicates kaempferol injection. Paired *t* test, *n* = 7. (**I**) Experimental scheme to assess the mitochondrial respiration of the VTA tissues. G, glutamate; P, pyruvate; M, malate; Scc, succinate; CCCP, mitochondrial oxidative phosphorylation uncoupler; Rot, rotenone; Ant, antimycin; C, complex; ER, endoplasmic reticulum; ETS max, maximal electron transport system capacity; NAD, nicotinamide adenine dinucleotide, oxidized form; NADH, nicotinamide adenine dinucleotide, reduced form. (**J**) Oxygen consumption rate of mitochondrial respiration in the VTA of mice with or without morphine EDA. 2-way RM ANOVA, *n* = 4–5 mice per group. Data are presented as mean ± SEM; *P < 0.05, ***P < 0.001, ****P < 0.0001.

Neurons depend on mitochondria not only to generate energy to maintain resting potentials and action potential firing, but also to regulate the balance of oxidation-reduction and calcium levels (33, 34). The level of nitrotyrosine, a marker for oxidative stress, was analyzed in the VTA dopaminergic neurons (Figure 3A). After morphine EDA, the nitrotyrosine level was significantly increased in the VTA dopaminergic Mor-Ens (TH^+^ tdTomato^+^), but not in nonensembles (TH^+^ tdTomato^–^) (Figure 3, B–D, and Supplemental Figure 4, A–C), suggesting toxic and oxidative damage in dopaminergic Mor-Ens.

Mitochondrial Ca^2+^ accumulation and efflux are critical for mitochondrial metabolism, ATP production, and cell death under pathological conditions (35). To measure neuronal mitochondrial Ca^2+^ dynamics in free moving mice, a strategy combining fiber photometry and intra-VTA injection of kaempferol was conducted (Supplemental Figure 4, D and E). *AAV-RAM-TTA-TRE-Flpo*, *AAV-fDIO-TH-Cre*, and *AAV-DIO-4mt-jGCaMP7b* viruses were injected into the VTA of *TH-Cre* transgenic mice (Figure 3E). Expression of the mitochondria-targeting Ca^2+^ sensor (mt-GCaMP7b) was restricted in the TH^+^ neurons and exhibited colocalization with mitochondrial marker COXIV (Supplemental Figure 4, F–H). Mitochondrial Ca^2+^ uptake is mainly mediated by the mitochondrial calcium uniporter (MCU) complex (36, 37). The mitochondrial Ca^2+^ signal in the VTA Mor-Ens was significantly increased by kaempferol, a MCU-specific activator that stimulates mitochondrial Ca^2+^ uptake (Figure 3F). The kaempferol-induced mitochondrial Ca^2+^ signal of the Mor-Ens was not significantly changed in the saline control group (Supplemental Figure 4, I–K), whereas it was significantly decreased by morphine EDA (Figure 3, F–H). High-resolution respirometry analysis in the VTA showed a significant decline in the complex I and II activity, as well as the maximal mitochondrial respiration, reflecting on a decreased oxygen consumption rate after Morphine EDA (Figure 3, I and J). These results show the increased oxidative stress and dysregulated Ca^2+^ transport in dopaminergic Mor-Ens, as well as impaired mitochondrial respiration in the VTA following chronic morphine administration.

**Figure 4:**
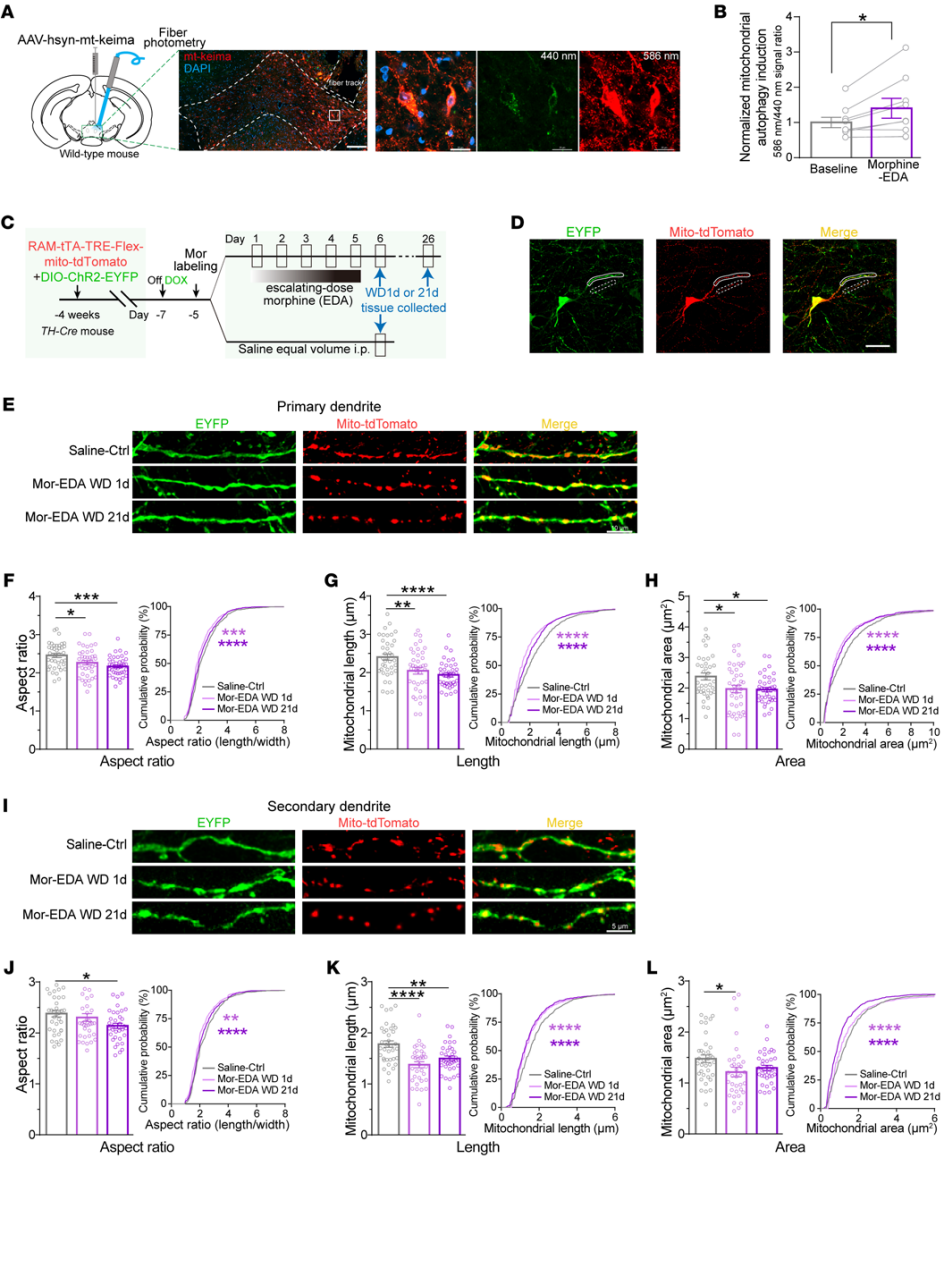
Chronic morphine administration increases the VTA neuronal mitophagy and mitochondrial fragmentation in the VTA dopaminergic Mor-Ens. (**A**) Experimental scheme for fiber photometry to detect mitophagy in Mor-Ens. Representative images show the expression of mt-keima in the VTA neurons. Dashed white lines outline the VTA and fiber optic tract. Scale bar, left: 200 μm; right: 20 μm. (**B**) Relative mitochondrial autophagy induction (% normalized to baseline) in the VTA neurons after morphine EDA. Paired *t* test, *n* = 9. (**C**) Experimental scheme to analyze mitochondrial morphology in Mor-Ens of mice in saline ctrl and morphine EDA groups. (**D**) Representative images of dopaminergic Mor-Ens expressing EYFP and mito-tdTomato. ChR2-EYFP was used to label dendrites and mito-tdTomato was used to label mitochondria. The white solid lines indicate primary dendrites and the dashed lines indicate secondary dendrites in each channel. Scale bar: 20 μm. (**E** and **I**) Representative images of primary dendrites (**E**) and secondary dendrites (**I**) containing labeled mitochondria from saline-ctrl, and withdrawal (WD) mice 1 day and 21 days after morphine EDA. Red, mito-tdTomato; Green, EYFP. Scale bars: 10 μm in **E**; 5 μm in **I**. (**F**–**H**) Quantification of mitochondrial aspect ratio (**F**), length (**G**), and area (**H**) in primary dendrites of dopaminergic Mor-Ens in saline-ctrl (38 neurons in 6 mice), morphine-EDA WD 1 day (40 neurons in 8 mice), or WD 21 day (40 neurons in 5 mice) groups. (**J**–**L**) Quantification of mitochondrial aspect ratio (**J**), length (**K**), and area (**L**) in dopaminergic Mor-Ens in saline-ctrl (36 neurons in 6 mice), morphine-EDA WD 1 day (35 neurons in 8 mice), or WD 21 day (38 neurons in 5 mice) groups. 1-way ANOVA with Bonferroni’s test and Kolmogorov-Smirnov test (**F**–**H** and **J**–**L**). Data are presented as mean ± SEM; *P < 0.05, **P < 0.01, ***P < 0.001, ****P < 0.0001.

The clearance of damaged mitochondria by mitophagy plays a fundamental role in mitochondrial function and metabolic homeostasis in neurons (38, 39). A mitochondria-targeting Keima (mt-Keima), a pH-sensitive dual-excitation fluorescent protein, was expressed in the VTA neurons. The mitophagy signal (the ratio of excited fluorescence intensity at 586 nm to 440 nm) in the VTA neurons was increased at 1 day after morphine EDA, but not changed in the saline control group (Figure 4, A and B, and Supplemental Figure 5, A and B). In addition, the increased mitophagy in the VTA neurons lasted up to 4 weeks after morphine EDA (Supplemental Figure 5, C and D). Mitochondria undergo dynamic processes including mitochondrial division, fusion, and elongation, which are critical for maintaining mitochondrial function and cellular quality (40, 41). *AAV-RAM-tTA-TRE-Flex-mt-tdTomato* and *AAV-DIO-ChR2-EYFP* were delivered into the VTA to label mitochondria in dopaminergic Mor-Ens (Supplemental Figure 5E). Morphology analysis of the tdTomato^+^ puncta in EYFP^+^ primary and secondary dendrites (Figure 4, C and D) showed the reduction of mitochondrial respect ratio, length, and area in VTA dopaminergic Mor-Ens at 1 day and 21 days after morphine EDA (Figure 4, E–L). Collectively, these results indicate that the mitochondrial fragmentation and impaired Ca^2+^ uptake in the VTA dopaminergic neurons, as well as impaired mitochondrial respiration and excessive neuronal mitophagy, are induced by chronic morphine administration.

### Restoration of the mitochondrial fusion gene Mfn1 in the VTA dopaminergic Mor-Ens alleviates oxidative stress and opiate withdrawal following chronic morphine administration.

**Figure 5:**
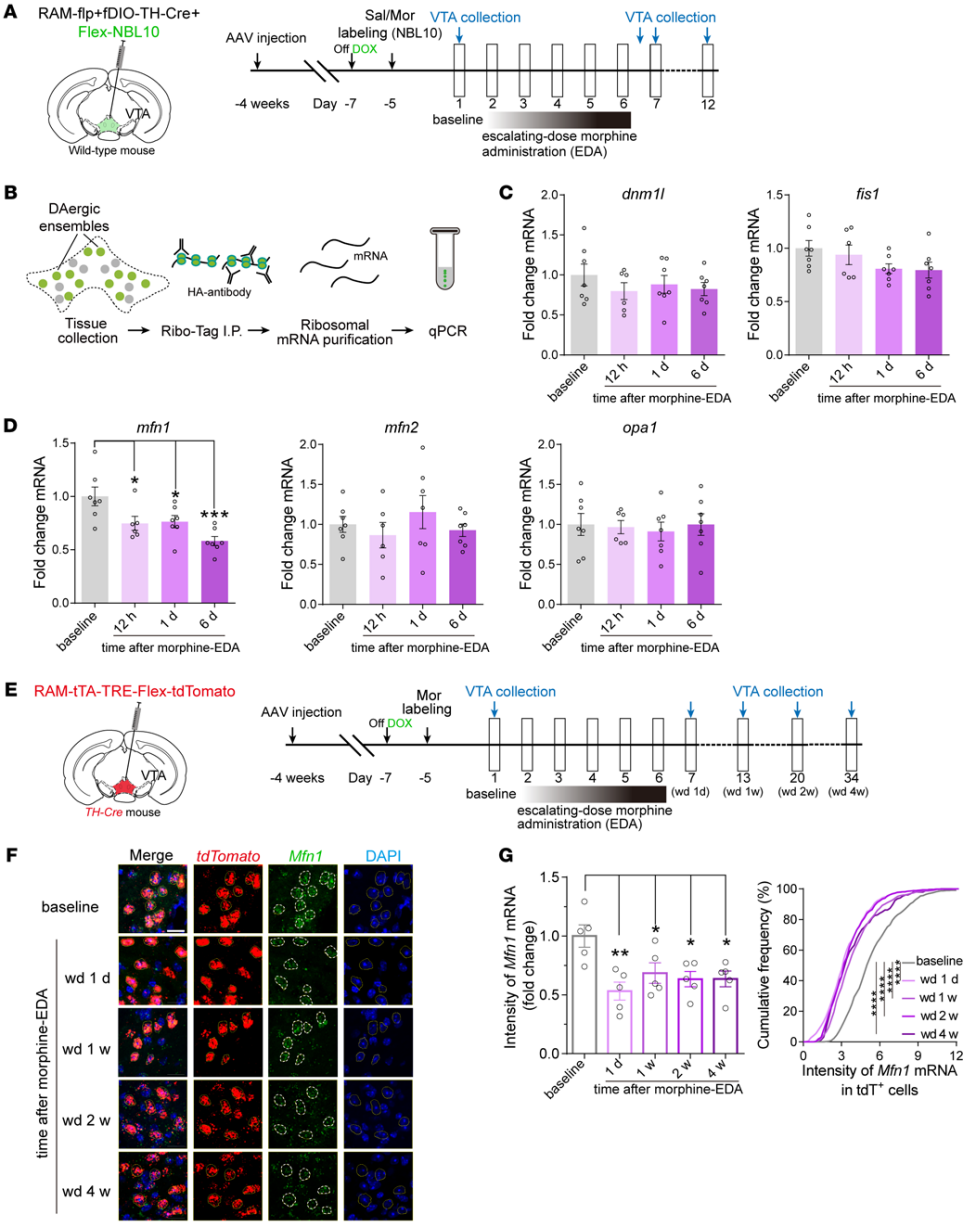
The expression of *Mfn1* is decreased in VTA dopaminergic Mor-Ens after chronic morphine administration. (**A** and **B**) Experimental scheme to label (**A**) and purify (**B**) the ribosome-associated mRNA from the dopaminergic ensembles expressing NBL10-HA. (**C** and **D**) Quantification of the relative mRNA levels of *dnm1l*, *fis1*, *mfn1*, *mfn2*, and *opa1* in VTA dopaminergic Mor-Ens at different time points after morphine EDA (normalized to the mice without morphine EDA). 6–7 mice per group, 1-way ANOVA with Bonferroni’s test. (**E**) Experimental scheme of the time line for VTA tissue collection. (**F**) Representative smFISH images of Mfn1 mRNA expressing in the VTA Mor-Ens at different time points after morphine EDA. Red, *tdTomato*; Green, *Mfn1*; Blue, DAPI. Dashed white lines outline the tdTomato^+^ cells. Scale bar: 20 μm. (**G**) Quantification of the *Mfn1* mRNA in the tdTomato^+^ Mor-Ens at different time points after morphine-EDA groups (normalized to the mice without morphine EDA). Cumulative frequency distribution of *Mfn1* mRNA intensity in the tdTomato^+^ Mor-Ens at different time points after morphine-EDA groups. *n* = 5 mice per group, baseline, 707 cells; wd 1 day, 743 cells; wd 1 week, 594 cells; wd 2 weeks, 669 cells; wd 4 weeks, 253 cells. 1-Way ANOVA with Bonferroni’s test, Kolmogorov-Smirnov test for cumulative frequency distribution. Data are presented as mean ± SEM; *P < 0.05, **P < 0.01, ***P < 0.001, ****P < 0.0001.

We then explored the potential signaling pathways involved in the mitochondrial dysfunction in VTA dopaminergic neurons. Mitofusin 1 (Mfn1) and Mfn2 mediate the fusion of mitochondrial outer membranes while optic atrophy 1 (Opa1) acts in the inner membrane. Dynamin 1 like (Dnm1l) and fission 1 protein (Fis1) mediate mitochondrial fission (42). Single cell–Seq data showed that the expression of these genes was not different between Sal-Ens and Mor-Ens, while *Mfn1* in Mor-Ens was downregulated after morphine EDA (Supplemental Figure 6, A and B). We then conducted a ribosome-associated mRNA pulldown experiment from VTA dopaminergic Sal-Ens and Mor-Ens, to investigate the temporal changes in mitochondrial functional dynamics induced by morphine EDA (Figure 5, A and B). Consistently, the mRNA levels of *Mfn1*, *Mfn2*, *Opa1*, *Dnm1l*, and *Fis1* were not different between Sal-Ens and Mor-Ens without morphine EDA (Supplemental Figure 6C). However, the mRNA levels of *mfn1* in the VTA dopaminergic Mor-Ens were decreased at 12 hours and the decrease persisted to 6 days after morphine EDA (Figure 5, C and D). In addition, single-molecule RNA fluorescence in situ hybridization (smFISH) was performed to detect the mRNA of *Mfn1* in the VTA dopaminergic Mor-Ens, and the results showed that the fluorescence intensity of Mfn1 mRNA in dopaminergic ensembles (tdTomato^+^) was decreased 1 day after morphine EDA and lasted up to 4 weeks after morphine EDA (Figure 5, E–G).

**Figure 6:**
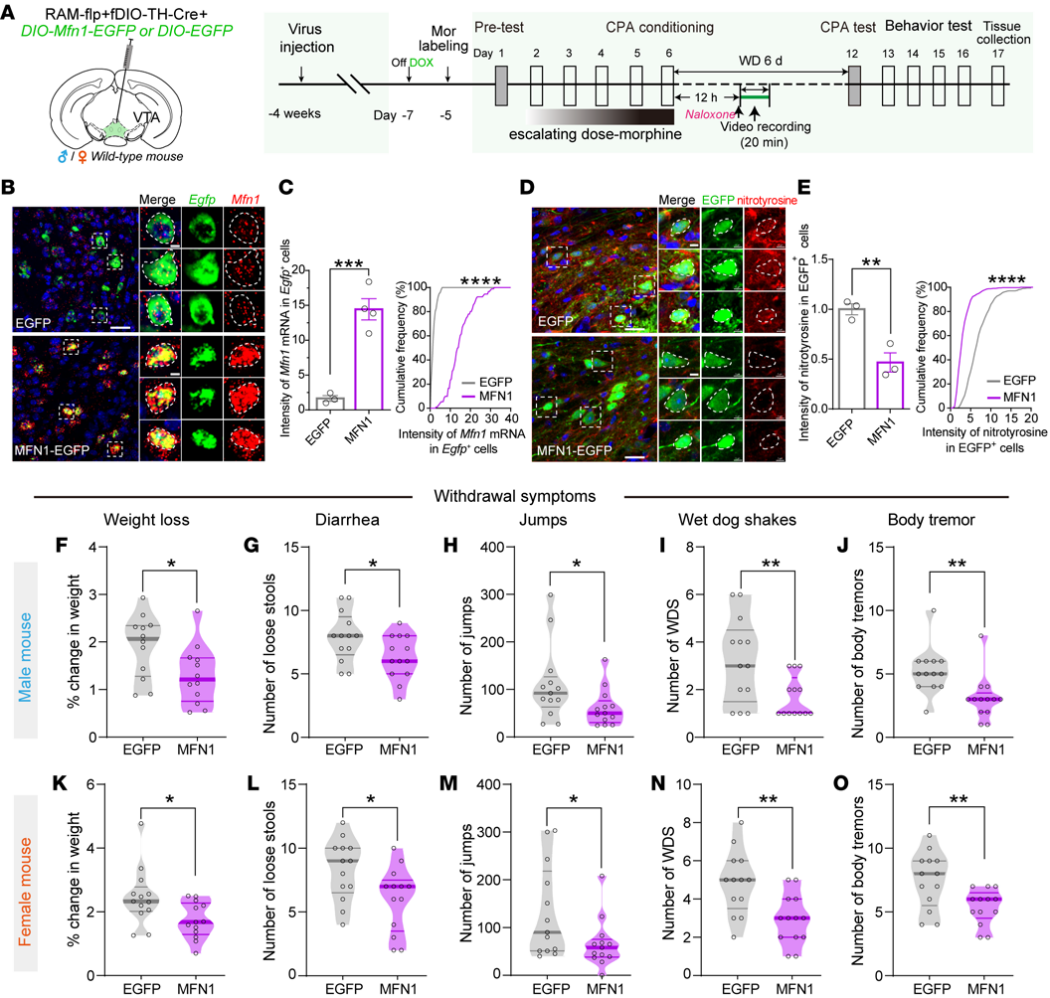
Overexpression of MFN1 in dopaminergic Mor-Ens alleviates withdrawal symptoms after chronic morphine administration in both male and female mice. (**A**) Experimental scheme to assess the effect of MFN1 overexpression in dopaminergic Mor-Ens. (**B**) Representative smFISH images of *Mfn1* mRNA expressed in the VTA Mor-Ens. Red, *Mfn1*; Green, *Egfp*; Blue, DAPI. Dashed white lines outline the *Egfp^+^* cells. Scale bars: 20 μm; 5 μm. (**C**) Quantification of the *Mfn1* mRNA in the *Egfp^+^* cells from EGFP and MFN1-EGFP groups. 3–4 mice per group, *Egfp*, 121 cells; *Mfn1*, 91 cells. (**D**) Representative images of nitrotyrosine immunostaining in the VTA Mor-Ens expressing EGFP or MFN1-EGFP. Red, nitrotyrosine; Green, EGFP; Blue, DAPI. Dashed white lines outline the EGFP^+^ cell. Scale bars: 20 μm; 5 μm. (**E**) The normalized expression level of nitrotyrosine in the VTA EGFP^+^ Mor-Ens. 3 mice per group, EGFP, 381 cells; MFN1, 434 cells. Unpaired *t* test or Kolmogorov-Smirnov test. (**F**–**O**) The effect of MFN1 overexpression in the VTA dopaminergic Mor-Ens on naloxone-precipitated withdrawal symptoms in both male and female mice. Weight loss, diarrhea, jumps, wet dog shakes, and body tremors were analyzed in EGFP and MFN1-EGFP groups. Male: 12–13 mice per group; female: 13 mice per group. Unpaired *t* test or Mann-Whitney test. Data are presented as mean ± SEM; *P < 0.05, **P < 0.01, ***P < 0.001, ****P < 0.0001.

To assess the involvement of Mfn1 in morphine withdrawal symptoms, mice were infected with *AAV-DIO-Mfn1-EGFP*, *RAM-tTA-TRE-flpo*, and *AAV-fDIO-TH-Cre* in the VTA to overexpress Mfn1 in dopaminergic Mor-Ens (Figure 6A). smFISH showed that the fluorescence intensity of *Mfn1* mRNA was dramatically increased in dopaminergic Mor-Ens (*Egfp^+^* cells) (Figure 6, B and C). Nitrotyrosine immunostaining showed that the restoration of MFN1-EGFP in dopaminergic Mor-Ens blunted nitrotyrosine induction in these ensembles after morphine EDA (Figure 6, D and E).

**Figure 7:**
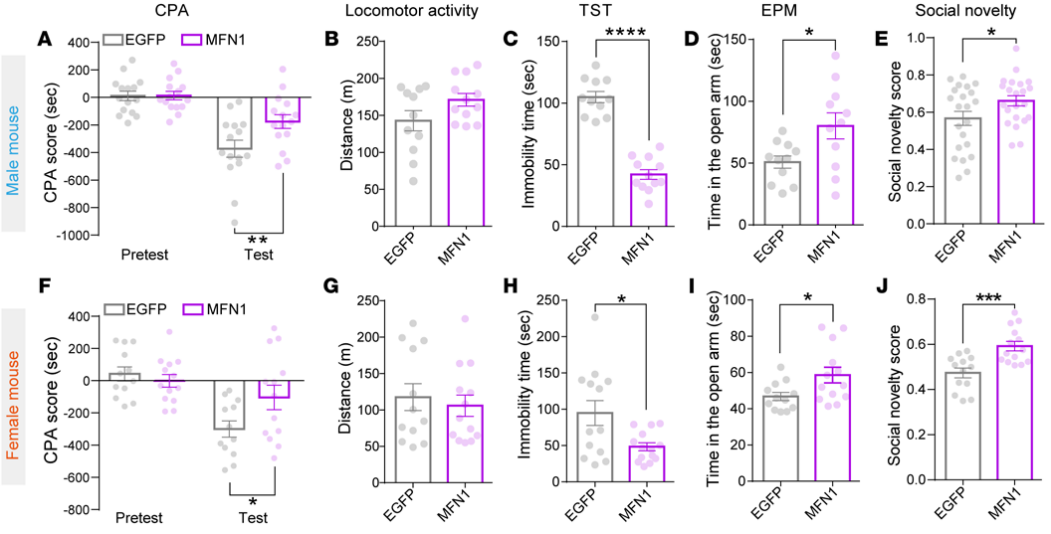
Restoration of MFN1 expression in dopaminergic Mor-Ens alleviates withdrawal-induced negative affects in both male and female mice. (**A**–**J**) The effect of MFN1 overexpression in Mor-Ens on negative affect during the spontaneous and prolonged morphine withdrawal in both male and female mice. Morphine withdrawal-induced CPA (**A** and **F**), locomotor activity (**B** and **G**), immobility time in TST test (**C** and **H**), time in the open arm in EPM test (**D** and **I**), and social novelty scores (**E** and **J**) were analyzed in EGFP and MFN1-EGFP groups. Male: 11–22 mice per group; female: 12–14 mice per group. Unpaired *t* test or Mann-Whitney test, 2-way RM ANOVA with Bonferroni’s test in CPA. Data are presented as mean ± SEM; *P < 0.05, **P < 0.01, ***P < 0.001, ****P < 0.0001.

In both male and female mice, overexpression of MFN1 in VTA dopaminergic Mor-Ens significantly reduced naloxone-precipitated morphine withdrawal symptoms, including weight loss, diarrhea, jumps, wet dog shakes, and body tremor compared with the EGFP control group (Figure 6, F–O), while piloerection was not affected. Backward locomotion was decreased by MFN1 only in females (Supplemental Figure 7, A and B). Overexpression of MFN1 in VTA dopaminergic Mor-Ens reduced spontaneous withdrawal-induced CPA score (Figure 7, A and F). Chronic morphine withdrawal produces negative affects, including anxiety, depression, and reduced sociability (43). Overexpression of MFN1 in the VTA dopaminergic Mor-Ens decreased immobility time in the tail suspension test (TST) (Figure 7, C and H) and increased open arm entry time in the EPM (Figure 7, D and I, and Supplemental Figure 7F) and social novelty score in the social interaction test (Figure 7, E and J and Supplemental Figure 7E), while not affecting locomotor activity (Figure 7, B and G) and social preference (Supplemental Figure 7, C and D) in both male and female mice.

These results demonstrate that downregulation of MFN1 in the VTA dopaminergic Mor-Ens contributes to the induction of withdrawal symptoms and negative affects, including anxiety, depression, and reduced sociability during opiate withdrawal. Cell type-specific manipulation of MFN1 may be a potential therapeutic strategy for opioid withdrawal in both male and female mice.

### Mdivi-1 ameliorates mitochondrial respiration of VTA, as well as mitochondrial fragmentation and dysregulated plasticity in the VTA dopaminergic Mor-Ens.

**Figure 8:**
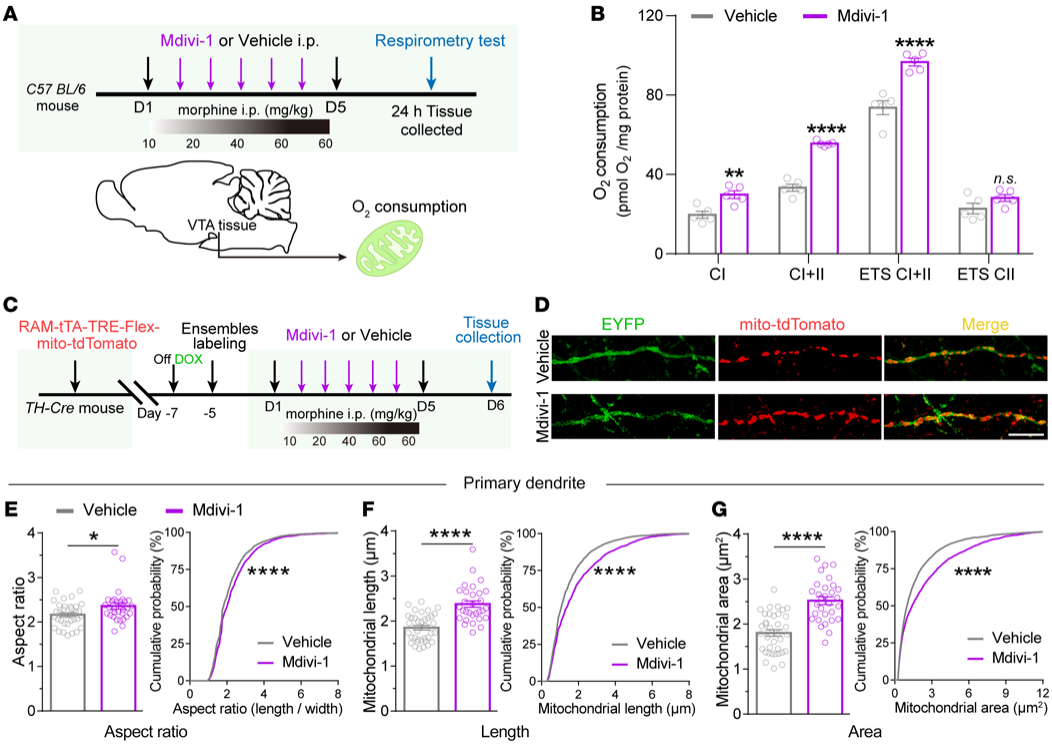
Mitochondrial division inhibitor Mdivi-1 restores the mitochondrial respiration of the VTA and ameliorates mitochondrial fragmentation in VTA dopaminergic Mor-Ens. (**A**) Experimental scheme to assess the mitochondrial respiration of VTA of mice in vehicle and Mdivi-1 groups. (**B**) Oxygen consumption rate of mitochondria in the VTA following morphine EDA in vehicle and Mdivi-1 groups. 5 mice per group, 2-way RM ANOVA by Bonferroni’s test. (**C**) Experimental scheme for tracing mitochondrial morphology in dopaminergic Mor-Ens after morphine EDA in mice of Mdivi-1 or vehicle groups. (**D**) Representative images of primary dendrites (green) containing mitochondria (red) after morphine EDA in Mdivi-1 or vehicle groups. Red, mito-tdTomato; Green, EYFP. Scale bar: 10 μm. **(E**–**G**) Quantification of mitochondrial aspect ratio (**E**), length (**F**), and area (**G**) in primary dendrites of dopaminergic Mor-Ens in vehicle (36 neurons in 6 mice) and Mdivi-1 groups (30 neurons in 8 mice). Unpaired *t* test and Kolmogorov-Smirnov test. Data are presented as mean ± SEM; n.s. not significant; *P < 0.05, **P < 0.01, ****P < 0.0001.

To explore pharmacological intervention targeting excessive mitochondrial fragmentation, Mdivi-1, a small molecule Mdivi-1 that selectively inhibits the mitochondrial division dynamin and blocks mitochondrial fission (44, 45), was administrated. Mitochondrial respiration of dissected VTA tissue from mice intraperitoneally injected with vehicle or Mdivi-1 (50 mg/kg, i.p.) was examined by high-resolution respirometry. Intraperitoneal injection of Mdivi-1 did not change VTA mitochondrial respiration in mice without morphine administration (Supplemental Figure 8, A and B). However, intraperitoneal injection of Mdivi-1 during the process of morphine EDA restored VTA mitochondrial respiration, as indicated by an increased oxygen consumption rate in mice receiving morphine EDA (Figure 8, A and B). The kaempferol-induced mitochondrial Ca^2+^ signal was not significantly different between Mdivi-1 and vehicle groups without morphine EDA (Supplemental Figure 8, C–E). However, the Mdivi-1 group exhibited higher kaempferol-induced mitochondrial Ca^2+^ signal in the VTA dopaminergic Mor-Ens than the vehicle control group after morphine EDA (Supplemental Figure 8, F and G). Without morphine EDA, intraperitoneal injection of Mdivi-1 moderately increased mitochondrial length and area in primary and secondary dendrites of the VTA dopaminergic Mor-Ens (Supplemental Figure 9, A–I). However, intraperitoneal injection of Mdivi-1 during the process of morphine EDA significantly increased the mitochondrial respect ratio, length, and area in the primary and secondary dendrites of the dopaminergic Mor-Ens (Figure 8, C–G, and Supplemental Figure 10, A–D). These results suggest that Mdivi-1 prevents mitochondrial fragmentation in dopaminergic Mor-Ens induced by morphine EDA.

**Figure 9:**
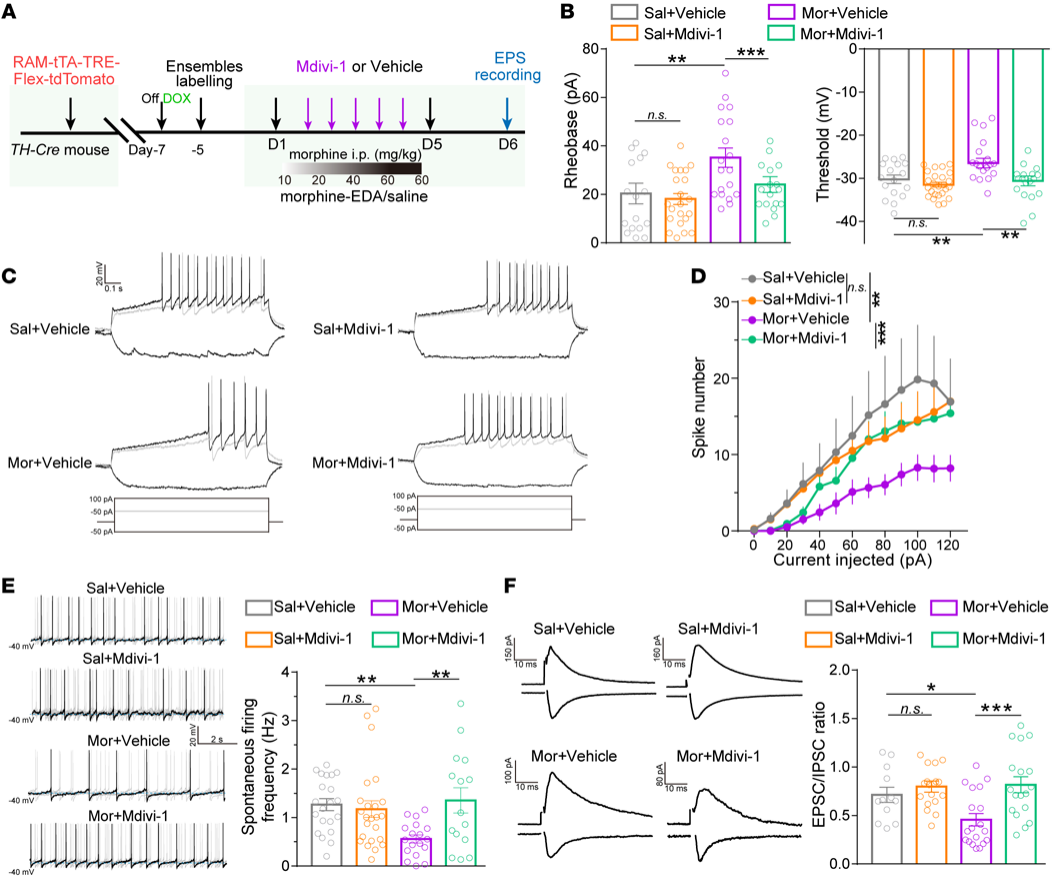
Mdivi-1 restores the maladaptation of neuronal plasticity in VTA dopaminergic Mor-Ens. (**A**) Experimental scheme of electrophysiological recording in dopaminergic Mor-Ens from saline and morphine EDA groups treated with vehicle or Mdivi-1. (**B**) Quantification of the rheobase and threshold of the action potentials in VTA tdTomato^+^ neurons. 4–5 mice per group; rheobase: 16–22 neurons per group, threshold: 17–24 neurons per group. (**C**) Representative AP traces and (**D**) quantification of the induced spike numbers of tdTomato^+^ neurons in the VTA. 13–18 neurons from 3–4 mice per group. (**E**) Representative traces and quantification of the spontaneous firing rate of tdTomato^+^ neurons in the VTA. 15–21 neurons from 3–4 mice per group. (**F**) Representative traces and quantification of the EPSC/IPSC ratio of tdTomato^+^ neurons in the VTA. 12–19 neurons from 3–4 mice per group. 2-way ANOVA by Bonferroni’s test in **B**, **E**,and **F**, 2-way RM ANOVA in **D**. Sal, saline; Mor, morphine. Data are presented as mean ± SEM; n.s. not significant; *P < 0.05, **P < 0.01, ***P < 0.001.

The effects of Mdivi-1 in the adaptations of neuronal plasticity in VTA dopaminergic Mor-Ens by chronic morphine administration were further investigated. Electrophysiological recordings were performed in tdTomato^+^ dopaminergic neurons (Figure 9A). Mdivi-1 administration had no effect on the spontaneous and evoked firing rate, as well as excitation-inhibition (E/I) ratio of these ensembles in the saline control group, whereas it prevented the increased rheobase and threshold of the of the action potentials, alleviated the downregulation of evoked spike number, spontaneous firing rate, and E/I ratio to restore regular firing in the dopaminergic Mor-Ens after morphine EDA (Figure 9, B–F). Neither morphine EDA nor Mdivi-1 injection had effect on the amplitude, half-width, and after-hyperpolarization potential of the VTA dopaminergic ensembles (Supplemental Figure 10E). These results demonstrate that Mdivi-1 ameliorates VTA mitochondrial respiration, inhibits excessive mitochondrial fission, and alleviates maladaptation of intrinsic excitability and plasticity of dopaminergic ensembles induced by chronic morphine administration.

### Mdivi-1 alleviates withdrawal symptoms and negative affects during morphine withdrawal in both male and female mice.

**Figure 10:**
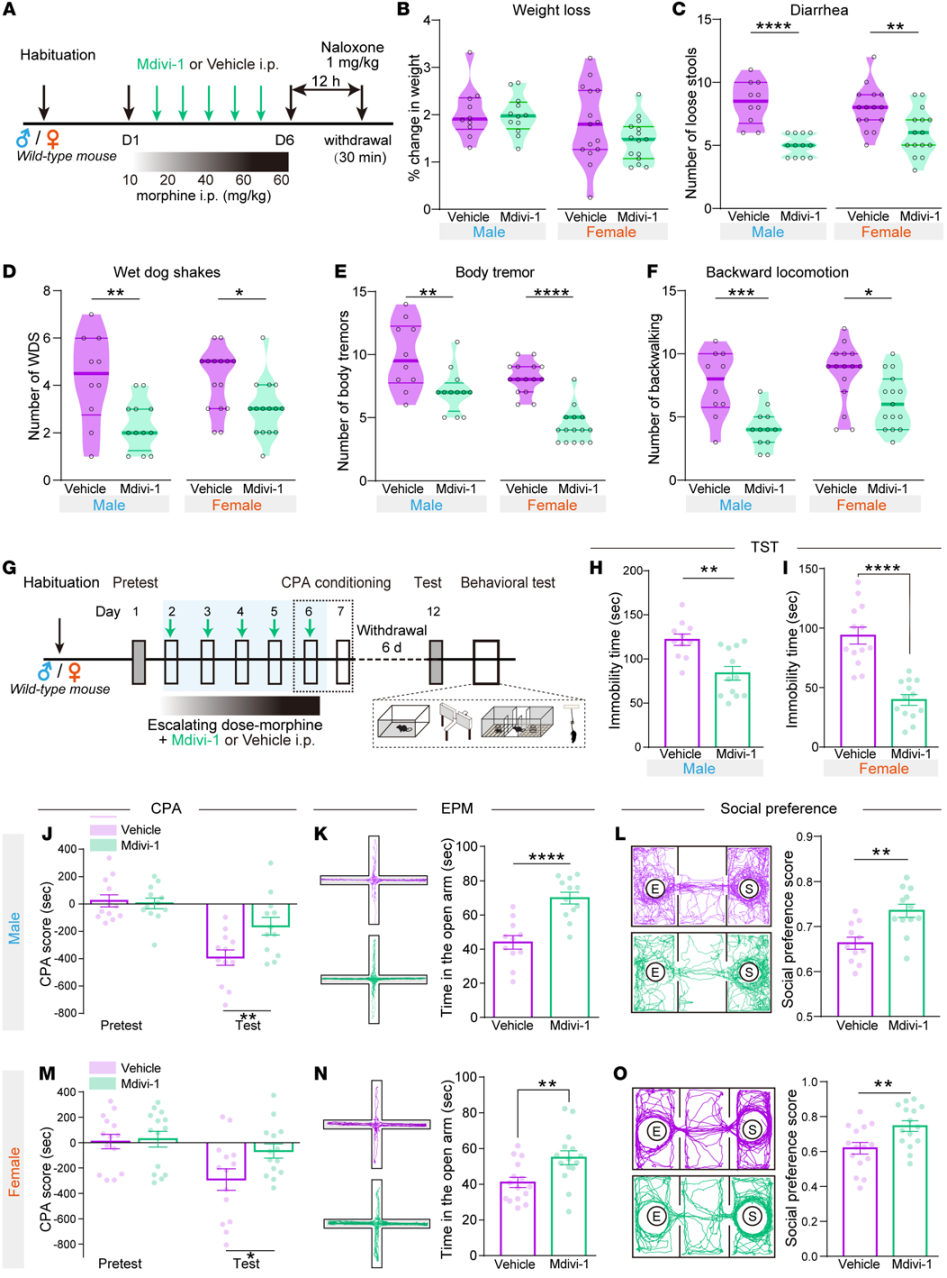
Mdivi-1 alleviates withdrawal symptoms and negative affects after chronic morphine administration in both male and female mice. (**A**) Experimental scheme to measure the naloxone-precipitated withdrawal symptoms in both male and female mice. (**B**–**F**) The effects of Mdivi-1 on weight loss (**B**), diarrhea (**C**), wet dog shakes (**D**), body tremor (**E**), and backward locomotion (**F**) were analyzed in mice from vehicle and Mdivi-1 groups. Male, 10-12 mice per group; female, 15 mice per group. Unpaired *t* test or Mann-Whitney test. (**G**–**O**) The effect of Mdivi-1 on negative affects during spontaneous and chronic morphine withdrawal in both male and female mice. (**G**) Experimental scheme of the behavioral tests. Immobility time (**H** and **I**), morphine withdrawal-induced CPA (**J** and **M**), time in the open arm (**K** and **N**), and social preference (**L** and **O**) were analyzed in vehicle and Mdivi-1 pretreated groups. Male, 11–12 mice per group; female, 14–15 mice per group. Unpaired *t* test or Mann-Whitney test for 2 groups, 2-way RM ANOVA with Bonferroni’s test in CPA. Data are presented as mean ± SEM; *P < 0.05, **P < 0.01, ***P < 0.001, ****P < 0.0001.

The effects of Mdivi-1 on naloxone-precipitated withdrawal symptoms and the negative affect during prolonged morphine withdrawal were measured in both male and female mice (Figure 10A). Both male and female mice received vehicle or Mdivi-1 during the process of escalated morphine injection. The number of diarrheas, wet dog shakes, body tremor, backward locomotion, and piloerection in Mdivi-1 group were decreased within 30 minutes after naloxone injection without affecting weight loss (Figure 10, B–F, and Supplemental Figure 11B), indicating that Mdivi-1 administration attenuated withdrawal symptoms. Whereas, the significant effect of Mdivi-1 on alleviating the number of jumps was observed only in the female mice (Supplemental Figure 11A). The Mdivi-1 group displayed a significant reduction in the spontaneous withdrawal-induced CPA score (Figure 10, J and M).

The negative affects in both male and female mice were assessed 6 days after the last injection of morphine EDA (Figure 10G). Mdivi-1 administration during the process of morphine EDA did not affect the total distance traveled in the open field tests (Supplemental Figure 11, C–F), while it significantly decreased the immobility time in the TST and increased the open arm entry time in the EPM (Figure 10, H, I, K, and N). In addition, Mdivi-1 administration increased the social preference during morphine withdrawal (Figure 10, L and O). These results suggest that Mdivi-1 administration during chronic morphine administration prevents withdrawal symptoms and negative affect during morphine withdrawal in both male and female mice.

### Mdivi-1 decreases the development of morphine-induced reinforcement and drug seeking after prolonged withdrawal.

**Figure 11:**
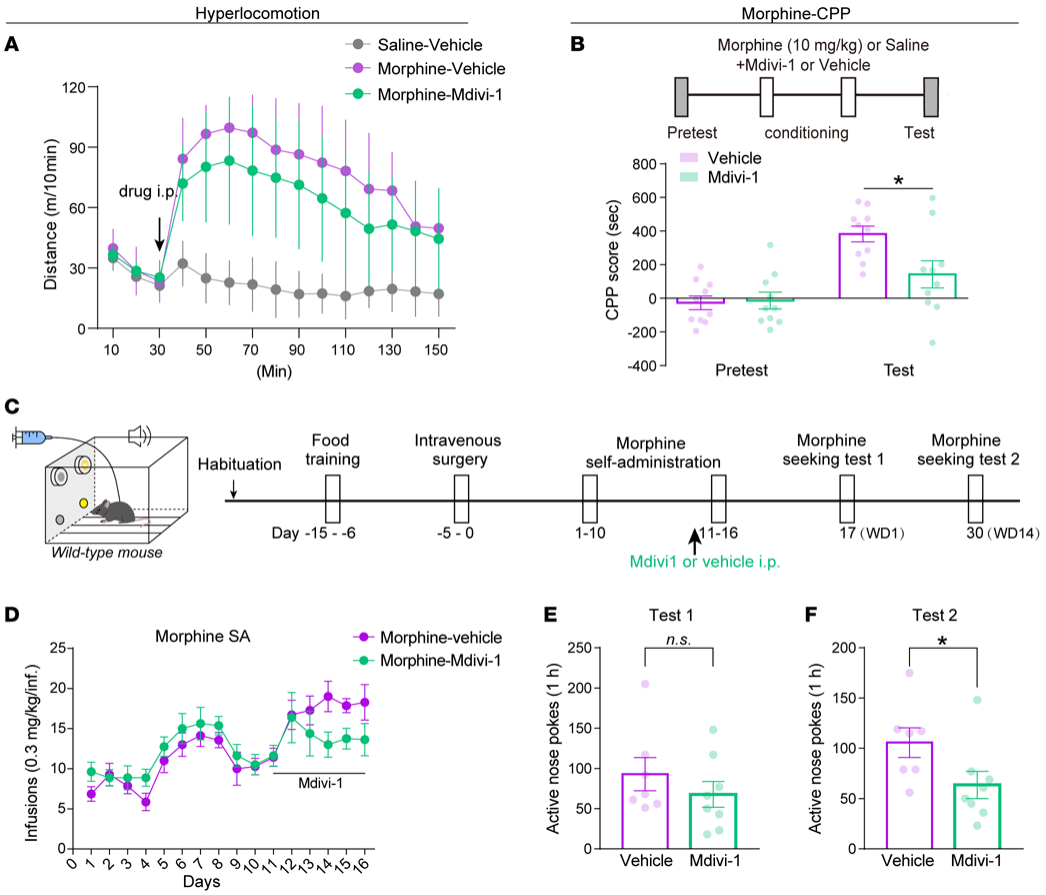
Mdivi-1 alleviates the development of morphine-induced reinforcement and drug seeking after prolonged withdrawal. (**A**) Morphine-induced hyperlocomotion in mice treated with Mdivi-1 (50 mg/kg, i.p.) or vehicle 45 minutes before the morphine injection. 10 mice per group, 2-way RM ANOVA. (**B**) Quantification of the morphine CPP scores in the mice injected with Mdivi-1 or vehicle 45 minutes before each conditioning session. 10 mice per group, 2-way RM ANOVA with Bonferroni’s test. (**C**) Experimental scheme to assess the effect of Mdivi-1 on drug seeking in a morphine SA paradigm. Mdivi-1 or vehicle was administrated during the 11–16 training sessions. (**D**) Numbers of morphine infusions in mice during the training sessions. (**E** and **F**) Plots of active nose pokes at 1 and 14 days after morphine withdrawal in mice from Mdivi-1 or vehicle groups. 7 to 8 mice per group, 2-way RM ANOVA, unpaired *t* test or Mann Whitney test. Data are presented as mean ± SEM; n.s. not significant; *P < 0.05.

Opioid analgesics induce reinforcement by activation of the mesolimbic dopaminergic reward circuits (46), which is an important contributing factor to the substance use disorders after prolonged opioid administration (47–50). In morphine-naive mice, Mdivi-1 conditioning did not establish appetitive or aversive place preference and had no effect on the anxiety level and locomotor activity (Supplemental Figure 12, A–C). The effect of Mdivi-1 on the rewarding properties of morphine was then further assessed. Mdivi-1 administration had no significant effect on morphine-induced hyperlocomotion (Figure 11A), however, the mice receiving Mdivi-1 or vehicle during the morphine conditioning resulted in decreased conditioned place preference (CPP) scores (Figure 11B), suggesting that Mdivi-1 attenuates the rewarding effect of morphine.

To assess whether Mdivi-1 could inhibit morphine-seeking behavior in the morphine self-administration (SA) paradigm, the mice that learned food SA by nose poke were randomly divided into 2 groups (Supplemental Figure 13A), and underwent intravenous catheter surgery and 16 days of morphine SA (0.3 mg/kg/infusion, FR1 schedule) (Figure 11C). Administration of Mdivi-1 at 11–16 days of SA had no effect on morphine acquisition (Figure 11D). 24 hours after the last session of morphine SA, the mice were returned to the chamber for the drug-seeking test. No difference in the number of active nose pokes was observed between the Mdivi-1 and vehicle groups (Figure 11E). However, the Mdivi-1 group displayed a significant reduction in the active nose pokes after 14 days of morphine withdrawal (Figure 11F), suggesting that Mdivi-1 alleviated drug seeking during prolonged withdrawal. These results reveal that Mdivi-1 administration during chronic morphine administration reduced the withdrawal symptoms and negative affects during morphine withdrawal and prevented the development of reinforcement and drug seeking behavior.

### Mdivi-1 alleviates the development of analgesic tolerance of morphine.

**Figure 12:**
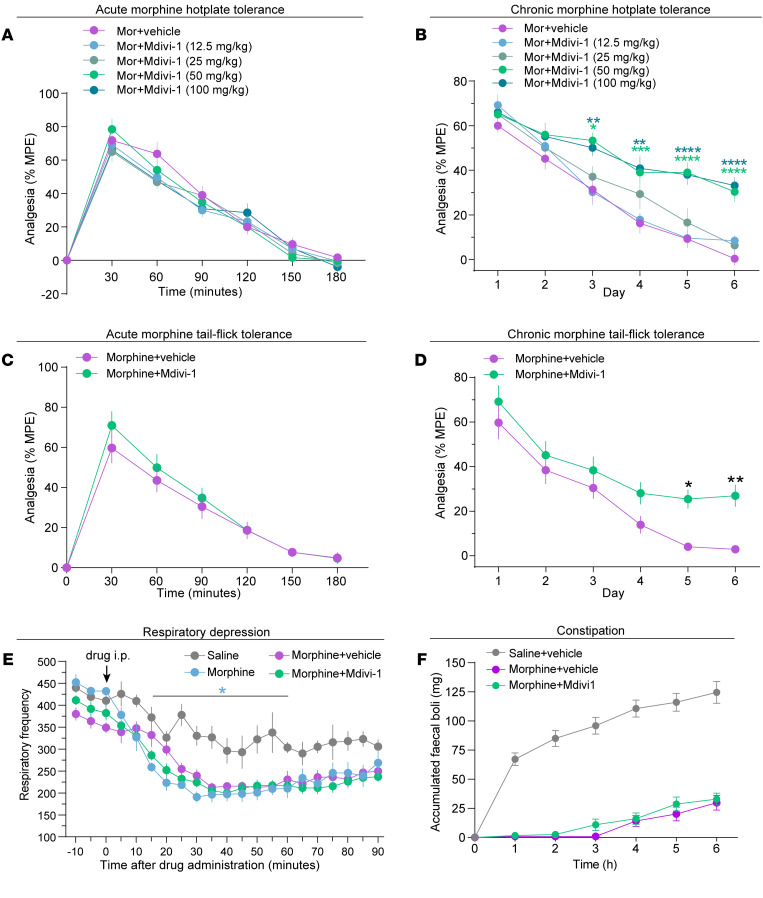
Mdivi-1 alleviates the development of analgesic tolerance of morphine. (**A**) The effect of Mdivi-1 on acute analgesia of morphine (10 mg/kg, i.p.) in hotplate assay. The latency of withdrawal to noxious stimulus is shown as the percentage of the maximum possible effect (% MPE). 12–13 mice per group. (**B**) The effect of Mdivi-1 on analgesic tolerance of chronic morphine (10 mg/kg, once daily) administration for 6 days. Analgesic tolerance was mirrored by the decreased % MPE. 12–15 mice per group, 2-way RM ANOVA with Bonferroni’s test for Morphine (Mor) + vehicle versus Morphine + Mdivi-1 (12.5, 25, 50, 100 mg/kg). (**C**) The effect of Mdivi-1 on acute analgesia of morphine (10 mg/kg, i.p.) in the tail flick assay. (**D**) Quantification of the analgesic tolerance of chronic morphine (10 mg/kg, once daily) in mice from Mdivi-1 (50 mg/kg) or vehicle groups. 12 mice/group, 2-way RM ANOVA with Bonferroni’s test in **C** and **D**. (**E**) Respiratory inhibition of morphine assessed by whole-body plethysmography in mice. Respiratory frequency is decreased 15 minutes after morphine injection (10 mg/kg, i.p). 2-way RM ANOVA by Bonferroni’s test, saline versus morphine, 4 mice/group; Morphine + vehicle versus Morphine + Mdivi-1, 8 mice per group. (**F**) Constipation effects of morphine assessed by accumulated faecal boli in Mdivi-1 or vehicle groups. 10–12 mice per group, 2-way RM ANOVA by Bonferroni’s test for saline + vehicle versus morphine + vehicle or morphine + vehicle versus Morphine + Mdivi-1. Data are presented as mean ± SEM; *P < 0.05, **P < 0.01, ***P < 0.001, ****P < 0.0001.

Prolonged exposure to morphine causes tolerance to analgesic effects, respiratory depression, constipation, and other side effects that limit the clinical use in the treatment of chronic pain (51, 52). Hot plate and tail flick tests were performed in WT mice to determine whether the analgesic effects of morphine and development of analgesic tolerance could be affected by Mdivi-1. The hot plate test assesses analgesia in both higher level central nervous system and spinal nociceptive circuits, while the tail flick test is more specific for spinal reflexive responses (53). Intraperitoneal injection of Mdivi-1 (12.5, 25, 50, and 100 mg/kg) did not affect the analgesic effects during 30 to 180 minutes after morphine (10 mg/kg) administration, as indicated by the similar maximum possible effect (MPE) curves between Mdivi-1 at different dosages and vehicle groups (Figure 12A). Repeated morphine (10 mg/kg) exposure over 6 days developed the tolerance of analgesic effects in the vehicle group. Whereas, the mice that receiving 50 and 100 mg/kg Mdivi-1 attenuated the development of morphine analgesic tolerance (Figure 12B). Consistently, Mdivi-1–pretreated (50 mg/kg) mice did not exert effects in the acute analgesia of morphine (10 mg/kg), while they did exhibit decreased morphine analgesic tolerance in the tail flick assay (Figure 12, C and D).

The effects of Mdivi-1 administration on morphine-induced respiratory depression and constipation were also investigated. Respiratory depression was assessed by whole-body plethysmography. Morphine profoundly depressed the respiration frequency compared with the saline group, while mice pretreated with Mdivi-1 exhibited an undistinguishable curve from the vehicle control group after morphine injection (Figure 12E). Mdivi-1 administration did not affect the constipating effect of morphine in mice, as indicated by the weight of accumulated fecal boli collected after morphine injection (Figure 12F). These results suggest that Mdivi-1 acts on both CNS and peripheral system to reduce the analgesic tolerance of morphine, while having no effect on morphine-induced respiratory depression and constipation.

## Discussion

**Figure 13:**
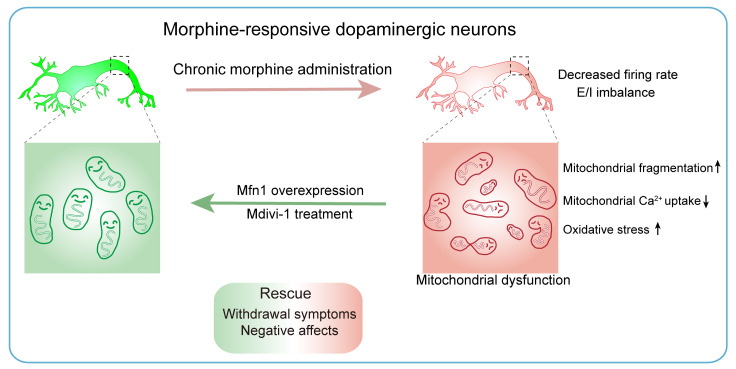
Strategy of targeting mitochondrial dynamics in morphine-responsive VTA dopaminergic ensembles to alleviate opiate withdrawal. Schematic diagram illustrating that chronic morphine administration decreases mitochondrial Ca^2+^ uptake and induces excessive fragmentation and oxidative stress in morphine-responsive VTA dopaminergic neurons, leading to the dysregulated mitochondrial respiration and maladaptation of the neuronal plasticity in the VTA. Genetic and pharmacological targeting the mitochondrial dynamics corrects mitochondrial fragmentation and neuronal plasticity in morphine-responsive VTA dopaminergic neurons and alleviates morphine withdrawal symptoms and negative affects.

This study provides evidence that mitochondrial dysfunction in opioid-sensitive dopaminergic neurons is involved in the development of opiate withdrawal. Our data suggest that chronic morphine administration decreases the activity, while increasing the mitochondrial fragmentation of VTA dopaminergic Mor-Ens, accompanied by low mitochondrial respiration in the VTA. Overexpression of Mfn1 in the dopaminergic ensembles or administration with Mdivi-1 restores the impaired mitochondrial function and regular firing of these ensembles, and attenuates the development of withdrawal symptoms and negative affect during opioid withdrawal (Figure 13).

Dysfunction of midbrain dopaminergic neurons is prominently implicated in chronic drug exposure. Opiate withdrawal markedly inhibits mesolimbic dopamine release, and animals experience a rebound aversive state after the acute reward is triggered (54). VTA dopaminergic neurons fire action potentials autonomously in a pacemaker pattern without presynaptic activation, which is often characterized by tonic activity (10, 11, 55). It serves to keep a steady background level of dopamine. There may be overlapping representations of VTA dopaminergic neurons of mice that respond to multiple stimuli (56). VTA dopaminergic neurons are spontaneously activated in home cage, and the volatile signal indicates the basal calcium activities probably representing a mixture signal of tonic firing in VTA dopaminergic ensembles in the home cage. The frequency of Ca^2+^ events in these ensembles decreased after chronic morphine administration, while activation of these ensembles during withdrawal alleviated the aversion and anxiety of the mice, suggesting the dysfunction of dopaminergic Mor-Ens involved in negative affects after chronic morphine administration.

Neurons are highly energy demanded and rely primarily on mitochondrial oxidative phosphorylation to provide ATP (57, 58). Mitochondria may directly sense the changes in the neuron under stimuli and stress conditions to shape adaptations (59). Recent studies demonstrate that mitochondria dysfunction occurs in neuropsychiatric diseases including major depressive disorders, anxiety, Alzheimer’s disease pathogenesis, progressive parkinsonism, bipolar disorder, and drug addiction (60–68). Disruption of mitochondrial complex I in dopaminergic neurons by deletion of *Ndufs2* is sufficient to cause progressive parkinsonism in which the loss of nigral dopamine release and slower or stopped pace-making of dopaminergic neurons mediate motor dysfunction (67). In our study, single-cell RNA-Seq analysis and mitochondria morphological analysis in these ensembles revealed the dysfunction and fragmentation of mitochondrial and excessive oxidative stress after chronic morphine administration.

Mitochondrial dynamics support energy generation, neurotransmitter release, and calcium buffering. The balance between fusion and fission is required to support the function of neuronal mitochondria, which drive diverse biological processes (32, 69). Imbalances in mitochondrial dynamics are associated with various diseases that are broadly characterized by impaired mitochondrial function and increased neuron death (70). Modulating mitochondrial fusion and division with either small molecules or genetic approaches has been implicated in the treatment of several brain disease models (71, 72). Inhibition of Drp1 effectively improved neuronal survival and function in several diseases characterized by excessive mitochondrial fragmentation (71). Drp1-mediated mitochondria fission in NAc D1-MSNs following repeated cocaine administration mediates drug seeking behavior during early abstinence (64). Drp1 inhibitor treatment restores the mitochondrial dynamics balance in the AD model and alleviates mitochondrial dysfunction associated with excessive β-amyloid–induced autophagy (73, 74). Reduced mitochondrial fusion is found in Fmr1-mutant mice, and enhancing mitochondrial fusion by compound M1, targeting MFN2, rescued dendritic abnormalities and behavioral deficits in these mice (75). The small molecule echinacoside and S89, which targets mitochondrial fusion progression, exert neuroprotective function in ischemic stroke (76, 77). In this study, the mitochondrial fusion gene Mfn1 was continuously downregulated in dopaminergic Mor-Ens following chronic morphine administration. Overexpression of MFN1 in these ensembles restores mitochondrial function and alleviates withdrawal symptoms and negative affects during morphine withdrawal. Besides, pretreatment with the mitochondrial fission inhibitor Mdivi-1 improves mitochondrial respiration in the VTA, restores mitochondrial Ca^2+^ uptake and alleviates mitochondrial fragmentation of the dopaminergic ensembles and withdrawal symptoms, suggesting that dysregulation of mitochondrial Ca^2+^ homeostasis and dynamics in VTA dopaminergic Mor-Ens are involved in the cellular and behavior maladaptation induced by chronic morphine exposure.

The toxic effects of morphine are not limited to the ensembles. Chronic opioid administration is associated with undesirable side effects, such as analgesic tolerance and adverse drug reactions (78, 79). The analgesic effect of opioids results from G_i_ signaling of the μ-opioid receptor, while side effects, including respiratory depression and constipation, may be conferred via the β-arrestin pathway. Agonists specific for the G_i_-biased μ-opioid receptor signaling pathway are thought to be the potential opioid analgesics with reduced side effects (53). In the present study, administration of Mdivi-1 did not exert effects in the acute analgesia of morphine or morphine-induced respiratory repression and constipation, while it did alleviate analgesic tolerance of morphine in both central nervous and peripheral systems, indicating that multiple in vivo targets of Mdivi-1 are involved in the therapeutic inventions after chronic morphine administration. In summary, this study demonstrates that mitochondrial dysfunction is an important cellular process in the brain after chronic morphine administration and is involved in the development of morphine withdrawal, providing potential therapeutic strategies targeting mitochondrial dynamics and homeostasis for opioid use disorders.

## Methods

### Animals

*Th-Cre B6.Cg-Tg(Th-cre)1Tmd/J* (Stock number: 008601) was obtained from the Jackson Laboratory, whose generation were bred on C57BL/6J background for more than 6 generations. Male offspring at 6–12 weeks of age were used in the experiments. Genotypes were determined by PCR of mouse tail DNA samples. C57BL/6 male and female mice aged 6–9 weeks were obtained from Shanghai Laboratory Animal Center (CAS). Mice used for experiments were housed in plastic cages with disposable bedding on a standard a 12 hour light/dark cycle with food and water available ad libitum. Experiments were performed during the light phase.

### Reagents

Morphine-hydrochloride (Shenyang 1st Pharmaceutical Co. LTD) and Clozapine-N-oxide (CNO, Sigma-Aldrich) were dissolved in saline. Mdivi-1 (Sigma-Aldrich) was dissolved in corn oil (Acros). Dox (MCE) was dissolved in water or food. All reagent information is listed in Supplemental Table 2.

### Viral constructs

Adeno-associated vector (AAV) *pAAV-RAM-d2TTA:TRE-FLEX-tdTomato-WPrepA* (Addgene: 84468), a cre-dependent RAM system, was used to label and manipulate specific subtypes of morphine–activated neurons. To generate the plasmids, *pAAV-RAM-d_2_TTA-pA:TRE-hM3D(Gq)-HA-WPrepA*, EGFP in *pAAV-RAM-d2TTA:TRE-EGFP-WPrepA* (Addgene: 84469) was replaced with hM3Dq(Gq)-HA, obtained by PCR from the templates of *pAAV-hSyn-DIO-(Gq)-mCherry* (Addgene: 44361). To generate the *pAAV-RAM-d_2_TTA-pA:TRE-Flpo-WPrepA* plasmid, *EGFP* in *pAAV-RAM-d2TTA:TRE-EGFP-WPrepA* (Addgene: 84469) was replaced with the *Flpo* sequence obtained by PCR from *pAAV-EF1*α*-Flpo* (Addgene: 55637). To generate the *pAAV- EF1*α*-fDIO-TH-Cre* plasmid, *hChR2(H134R)-EYFP* in *pAAV-Ef1*α*-fDIO-hChR2(H134R)-EYFP* (Addgene: 55639) was replaced with the *TH-Cre* sequence obtained by PCR from *pAAV.rTH.PI.Cre.SV40* (Addgene: 107788). To generate the *pAAV-EF1*α*-DIO-Mfn1-EYFP* plasmid, *Mfn1* sequence obtained by PCR from *pMfn1-Myc* (Addgene: 23212) was inserted in *pAAV-Ef1*α*-DIO-EGFP* (Addgene: 27056).

For mitochondrial tracing, the mitochondrial targeting (mt) sequence from cytochrome oxidase subunit 8A (COX8A) was fused to the N-terminus of *tdTomato* to generate *pAAV-RAM-d2TTA:TRE-FLEX-mito-tdTomato-WPrepA*. To generate the *pAAV-DIO-4mt-GCaMP7b* plasmid, 4 repeated mt sequences were inserted at the N-terminus of *GCaMP7b* in *pAAV-DIO-GCaMP7b* (purchased from BrainVTA Co. Ltd). All viruses described above were packed into AAV serotype 9 (Obio technology Co., Ltd). *AAV-DIO-ChR2-EYFP*, *AAV-DIO-EGFP*, and *AAV-EF1*α*-FLEX-NBL10* were purchased from Taitool Biological Co., Ltd (Shanghai, China). *AAV-hsyn-mt-keima* was purchased from WZ Biosciences Co. Ltd. AAV preparations with a titer 2 × 10^12^ TU/mL were used. All primer sequences are listed in Supplemental Table 1.

### Morphine- or saline-recruited neuronal ensembles labelling

After surgery, mice were kept on a Dox-containing diet (1 g/kg) for 2–3 weeks. For labelling activated neuronal populations, mice were taken off of the Dox diet for 48 hours before intraperitoneally injection with 10 mg/kg morphine or saline, and then kept on a Dox-containing regular diet or Dox water (200 mg/kg) for 8 hours after the injection to stop further labeling. Mice were given 3–5 days for recovery before the behavior tests, single-cell analysis, fiber photometry recording, and electrophysiological recording.

### In vivo fiber photometry recording

Fluorescence signals in neurons were recorded using a fiber optic photometry system equipped with 470- and 410-nm excitation lasers (Inper Tech). The laser power at the tip of the optical fiber was adjusted to 10 to 15 μW for 410 nm and 25–30 μW for 470 nm by optical power meter (Thorlabs, PM100D). 470 nm (Ca^2+^-dependent) and 410 nm (isosbestic reference fluorescence) fluorescence signals were collected by the MiniCMOS at 50 Hz for single channel. Each mouse was detected for 1 trial at 1 hour before (baseline) and 1 day after morphine EDA. The raw signals were adjusted to a flat baseline after baseline and motion correction using a script provided by Inper Tech; the baseline-adjusted signals were transformed as ΔF/F by dividing by their mean raw signals. And then a value of 4 times the median absolute deviation (4 × MAD) of ΔF/F of the baseline session was used as the threshold for event detection with a value of 90% of 4 × MAD to rearm event detection (15, 80).

For recording and analysis of the mitochondrial Ca^2+^ signals, the mitochondria-targeted Ca^2+^ sensor GCaMP (Mito-GCaMP) (59, 81) was expressed in VTA dopaminergic ensembles. Mice were infused with 1.6 μL of saline or kaempferol (2 nmol/μL; Sigma-Aldrich, VTA injection) into the VTA at a slow injection rate of 0.2 μL/min controlled by a microinjection pump (WPI). The fluorescence signals were recorded by Inper Tech. The 470 nm and 410 nm signals were collected separately and normalized to baseline signals to determine Δ*F/F*. Δ*F/F* = (*F–F*_0_)/*F* where *F*_0_ is the mean value of the integrated prestimulus signal (100 s). GCaMP signals were analyzed and plotted with MATLAB R2019b (MathWorks) as previously described (18). Δ*F/F* values are presented as heatmaps and average plots, with the shaded area indicating the SEM.

To monitor and measure mitophagy, a 3-color fiber photometry equipped with 410-, 470-, and 570-nm excitation lasers (Inper Tech) was used to measure mitophagy. The mitochondrial targeting Keima, which is resistant to lysosomal proteases and has bimodal excitation spectrum (440 and 586 nm) that depends on the surrounding pH, was expressed in the VTA. Shorter wavelength excitation predominates at the physiological pH of mitochondria (pH 8.0), whereas Keima undergoes a gradual shift to longer wavelength excitation in the lysosome (pH 4.5) after mitophagy (82–84). Mitophagy induction is equal to signals excited at 586 nm divided by signals excited at 440 nm. Each mouse was detected for 1 trial 1 hour before and 1 day, 2 weeks,or 4 weeks after morphine EDA in the home cage. Raw signals were adjusted to a flat baseline after baseline and motion corrections by Inper Tech; the baseline-adjusted signals were transformed to ΔF/F by dividing by its mean raw signals. Relative mitochondrial autophagy induction was normalized to baseline. Mice with off-target fiber tips were excluded from the analysis.

### Behavioral experiments

#### Morphine withdrawal-induced CPA.

CPA conditioning was performed as previously described (18). Briefly, mice were allowed to freely explore both sides of 2 chamber training apparatus (Med-Associates) for 20 minutes (pretest) and then divided into 2 groups for labeling of neuronal ensembles. Mice received escalating doses of morphine injection (10, 20, 40, 60, and 60 mg/kg) every 12 hours (10:00 am; 22:00 pm) for 5 consecutive days in the home cage to establish morphine dependence. Nine hours after each of the last 3 morphine injections, when the spontaneous withdrawal is induced, mice were confined in 1 chamber (withdrawal-paired) of the apparatus for 30 minutes. Posttests took place 6 days after the last conditioning trial. Mice were reexposed to chamber for 20 minutes. The time spent in each chamber was recorded. The CPA score was defined as the time spent in the withdrawal-paired chamber minus the time spent in the other side of the chamber. For chemogenetic manipulations, mice received an injection of CNO (2 mg/kg, i.p.) 30 minutes before each of the CPA conditioning tests. To assess the effect of Mdivi-1 on morphine-withdrawal CPA, the mice were pretreated with Mdivi-1 or vehicle for 45 minutes before each morphine injection.

#### Assessment of withdrawal symptoms.

To measure withdrawal symptoms, mice were injected with an escalating dose of morphine (10, 20, 40, 60, 60 mg/kg) twice daily for 5 consecutive days. Withdrawal was precipitated by naloxone (1 mg/kg, i.p) 12 hours after the last morphine injection. The withdrawal symptoms of each mouse were recorded for 30 minutes by a 30 FPS camera in the home cage. Mice were weighted before and after naloxone injection, and the weight loss was calculated as the percentage of the initial weight. Diarrhea was measured by the number of accumulated faecal boli. Withdrawal symptoms (jump, wet dog shake, body tremor, backward locomotion, and piloerection) analysis were performed by an individual blinded to group assignment.

### Measurement of morphine analgesia and tolerance

Analgesia-like responses in mice were measured using a hotplate analgesia meter (Columbus Instruments) and a radiant heat tail-flick meter (UGO basile) as previously described (53, 63). For morphine hot-plate tolerance, repeated morphine injections (10 mg/kg, i.p.) were administrated daily for 6 days. The hot-plate test was performed on a platform heated to 50°C with a cut-off of 60 seconds, and the latency to lick paws or splay hind paws or jump was recorded. Baseline response was determined for each mouse before treatment. Once a response was observed or the cut-off time had elapsed, the mouse was immediately removed from the hotplate and returned to its home cage. For the tail flick test, mice were restricted in plexiglas cages on a modified platform. Mice were habituated to the device for 2 minutes before each test session. The infrared heat stimulus was focused on mouse tail and the response time of tail flicks was automatically determined by a sensor. A 30 second cut-off time was used to avoid tail damaging. Mice were preinjected with vehicle or Mdivi-1 (12.5, 25, 50, and 100 mg/kg) 45 minutes before morphine injection. After injection of morphine (10 mg/kg), the analgesic effect was measured at 30, 60, 90, 120, 150, and 180 minutes after injection. The analgesic tolerance response to morphine was assessed by the radiant heat tail-flick test or hot-plate test at 30 minutes after morphine injection. The analgesic effect was calculated as %MPE; MPE % = (test latency - baseline latency)/(cut-off time-baseline latency) × 100.

### Mouse plethysmography

Respiration data was detected using a whole-body plethysmography system (TOW-INT TECH Inc.), as described previously (53). Respiratory frequency, tidal volume, and peak flows were measured in unrestrained mice. Airflow transducers were attached to each plethysmography chamber maintained at a constant flow rate. Each chamber was calibrated to its attached transducer before the experiment. Mice were habituated to the test chambers for 30 minutes. Respiratory parameters were recorded for 10 minutes to establish a baseline before injection of saline or morphine (10 mg/kg). Mice were preinjected with vehicle or Mdivi-1 (50 mg/kg) 45 minutes before morphine injection. Respiratory parameters were then collected from unrestrained mice for 90 minutes after morphine injection.

### Mitochondrial respirometry

Mice were sacrificed and the VTA tissues were rapidly dissected out, weighed, and placed in a petri dish on ice with 2 mL of BIOPS relaxing solution (2.77 mM Ca_2_K_2_EGTA, 7.23 mM K_2_EGTA, 5.77 mM Na_2_ATP, 6.56 mM MgCl_2_, 20 mM taurine, 15 mM sodium phosphocreatine, 20 mM imidazole, 0.5 mM dithiothreitol and 50 mM MES, pH = 7.1 [all from Sigma-Aldrich]) and gently homogenized with an eppendorf pellet pestle in ice-cold respirometry medium (MiR05: 0.5 mM EGTA, 3mM MgCl_2_, 60 mM potassium lactobionate, 20 mM taurine, 10 mM KH_2_PO_4_, 20 mM HEPES, 110 mM sucrose and 0.1% (w/v) BSA, pH = 7.1 [all from Sigma-Aldrich]). The high resolution respirometry instrument (Oxygraph-2k, OROBOROS Instruments) was used to detect mitochondrial respiration rates at 37°C, as previously described (62, 72). A sequential multisubstrate protocol was used to explore the individual components of mitochondrial respiration capacity. Oxygen flux due to complex I activity (Complex I) was quantified by adding ADP (2 mM) to a mixture of 0.8 mM malate,4 mM pyruvate, and 8 mM glutamate. Succinate (8 mM) was added sequentially to reconstitute convergent complex II (Complex I + II) respiration. Titrations with the uncoupler CCCP (0.4 μM) were performed to determine electron transfer system (ETS) capacity. Rotenone (0.08 μM; ETS CII), which could inhibit complex I, was added to examine consumption in the uncoupled state due to complex II activity alone. Electron transport through complex III was inhibited by adding antimycin (2 μM) to obtain the level of residual oxygen consumption (ROX). The O_2_ flux obtained in each step of the protocol was normalized by the wet weight of the tissue used for the analysis.

### Mitochondrial imaging and analysis

Images of the neuronal ensembles in the VTA were taken by a confocal microscope (Nikon-1A). High-resolution Z-stack images were obtained with 0.5 mm increments using a × 20 air objective with × 2 digital zoom. Neuronal reconstruction and maximum intensity projection were performed with the automatic deconvolution (NIS-Elements AR 5.02.00). 3D reconstructions of the images were generated from each channel, red (mitochondria) and green (soma and dendrites). Mitochondria aspect ratio, length, and area were analyzed by using Image-pro Plus 6.0 software. The location of primary (50 μm from soma) and secondary (branch from the primary dendrite) dendrites was identified. The tdTomato^+^ mitochondria identified within the dendrites were analyzed. Single mitochondrion were identified by the grayscale ranged 162 to 255 and the area ranged 5 to 200 pixels. The mitochondrial aspect ratio, length, and area were measured and converted to the measurement scale (169 pixels = 50 μm).

### Statistics

Data were analyzed with SPSS software (IBM). Sample sizes were based on our previous research (15, 18, 26). The normality test of the data was performed by Shapiro-Wilk test and the homoscedasticity was performed by F test. Kolmogorov-Smirnov test was used for analyzing the cumulative distribution. Comparisons between groups were made by Student’s *t* test (Unpaired, 2-tailed), Mann-Whitney U test, or 1-way ANOVA. 2-way ANOVA and 2-way repeated measure (RM) ANOVA were used followed by Bonferroni’s post hoc test. Statistical significance was represented as *P < 0.05; **P < 0.01; ***P < 0.001; and ****P < 0.0001. All data are presented as mean ± SEM.

### Study approval

All animal procedures followed the animal care guidelines approved by the Animal Care and Use Committee of Shanghai Medical College of Fudan University. The Ethics Committee of Shanghai Medical College, Fudan University, approved the study protocol.

### Data and code availability

Sequencing data have been deposited in the Gene Expression Omnibus under accession number PRJNA949982, and all analyses were performed using existing packages. Values for all data points in the plots are provided in the Supporting Data Values file.

## Author contributions

LM and FW supervised the study. CJ contributed to the experimental design, statistical analysis, and drafting of the manuscript. CJ, HH, and XY, performed the surgery, behavioral and molecular experiments. CJ conducted the electrophysiological recordings, image acquisition, and data analysis. QL and CJ conducted the RNA-Seq and bioinformatics analysis. LM, FW, CJ, QL, and XL revised the manuscript.

## Supplementary Material

Supplemental data

Supporting data values

## Version 1 01/18/2024

In-Press Preview

## Version 2 03/01/2024

Electronic publication
