# Supplementary material for: Targeting mitochondrial dynamics of morphine-responsive dopaminergic neurons ameliorates opiate withdrawal
Source: J Clin Invest. 2024 Jan 18;134(5):e171995. doi: 10.1172/JCI171995 (PMC10904060; doi:10.1172/JCI171995)
Supplement: Supplemental data [file jci-134-171995-s245.pdf]

SUPPLEMENTARY MATERIAL

SUPPLEMENTARY METHOD

Stereotaxic surgery

All surgeries were performed under stereotaxic guidance and the coordinates are given relative to bregma. Mice were anesthetized with isoflurane (3.5% induction, 1.5-2% maintenance, RWD, China) and placed in a stereotactic instrument (Stoelting, Kiel, WI, USA). AVV virus microinjections were performed with 33-gauge needles connected to a 10 μL Hamilton syringe (Hamilton, Bonaduz, Switzerland). Viruses were delivered with a slow injection rate of 0.2 μL/min into the ventral tegmental area (VTA) (AP − 3.1 mm; ML ± 0.5 mm; DV − 4.5 mm). Mice were kept on a heating pad until recovered from the surgery. For the in vivo photometry recording, an optical fiber with an outer diameter of 400 μm and 0.50 numerical aperture (Inper Tech, Hangzhou, Zhejiang, China) was unilaterally implanted into the VTA of mice (AP − 3.1 mm; ML ± 0.5 mm; DV − 4.1 mm). For intracerebral injection in VTA, cannula guides were unilaterally implanted (Plastics one, Roanoke, VA, USA) above the VTA of mice (AP − 3.1 mm; ML ± 0.5 mm; DV – 3.0 mm). The cannulas or optic fibers were secured to the skull of mice with dental cement. All mice were allowed 2-3 weeks to allow for recovery and maximal viral expression before behavioural experiments.

Behavioral experiments

Conditioned place preference conditioning

Conditioned place preference (CPP) was performed blindly by an investigator using the two-chamber apparatus with different tactile contexts (Med-Associates, USA). On day 1, mice were allowed to freely explore the apparatus for 20 min (pre-test). Mice that stayed in one chamber for more than 13

min were excluded. On days 2 and 3, mice received an intraperitoneal injection of morphine (10 mg/kg) and were confined to one of the chambers (drug-paired) for 30min (conditioning). 6 hrs later, they received an i.p. injection of saline and were confined to the other chamber for 30 min. On day 4, mice were allowed to freely explore the entire apparatus for 20 min (test). The time spent in each chamber was recorded during the pre-test and test sessions. To determine the effect of Mdivi-1 on morphine CPP, the mice were injected with Mdivi-1 or vehicle 45 min prior to morphine conditioning sessions.

Three-chamber social preference test

The social preference test was performed in the apparatus divided into three chambers (40 cm length ×22 cm width × 23 cm height) with two empty wire mesh cups placed in two opposite corners. The experimental mice were habituated in the apparatus for 10 min. During the social interaction test, a novel (unfamiliar) mouse (4-6 weeks old) was placed in one of the wire mesh cups to prevent physical contact, while the other chamber contained the empty wire cage. The test mouse was placed in the middle chamber and allowed to explore the entire chamber. For the social novelty test, the familiar mouse (from the previous social interaction phase) was placed in its original wire cage, and a novel unfamiliar mouse was placed in the wire cage in the other chamber. All the sessions were recorded for 10 min with a digital video camera and analyzed by video tracking software (Noldus). Interaction behaviors included proximity (within 2 cm), standing, sniffing, nose-to-nose contact, *etc*. The box and wire cage were cleaned with 75% ethanol between trials. Social preference score = time in unfamiliar mouse side/ (time in unfamiliar mouse side + time in empty side). Social novelty score = time in unfamiliar mouse side/ (time in unfamiliar mouse side + time in familiar mouse side).

Open field, elevated plus maze, and tail suspension tests

Mice were allowed to freely explore the open field chamber (40 cm length × 40 cm width × 30 cm height) for 30 min (baseline) before intraperitoneal injection of morphine (10 mg/kg), and then allowed

to explore the open field for another 120 min. Activity was recorded at 5-min or 10-min intervals by means. Entries to the central zone, time spent in the central zone and total distance traveled were analyzed by the video tracking program (Noldus, Wageningen, Netherlands).

The elevated plus maze consisted of four arms (34.5 cm length × 6.3 cm width × 19.5 cm height) and a central platform placed 75 cm above the floor in a quiet and dimmed room. Mice were placed in the center and allowed to move freely for 6 min. Their behaviors were recorded by a camera placed above the maze. The location, velocity and movement of the head, body and tail were analyzed by the video tracking program (Noldus). Time spent in open arms was assessed.

The tail of the mouse was secured with a tape at 1.5-2 cm from the end, and then suspended upside down on a crossbar 15-20 cm above the floor without any accessible surface for 6 min. Videos were recorded for trials to analyze the cumulative time of struggling and immobility within 6 min. For chemogenetic manipulations, mice received an intraperitoneal injection of CNO (2 mg/kg, i.p.) 30 min before the behavioral tests. The apparatus was cleaned with 75% ethanol and dried between each trial to ensure the absence of olfactory cues.

Intravenous morphine self-administration (SA)

Mice were placed in operant conditioning chambers (ENV-307W-CT, Med Associates, USA) and trained to nose poke for food pellets (14 mg, catalog #F05684, Bio-serv, USA) under a fixed ratio (FR5 to FR 20) reinforcement schedule for 10 days. Mice that met the criteria (> 20 food rewards in one session, 80% accuracy between nose pokes) were implanted with an indwelling jugular catheter (#781-43-2202, Braintree Scientific Inc., USA). The catheter was subcutaneously threaded from the jugular vein to the back, and exited the body via a guide cannula. Mice were individually housed and flushed daily with 0.1 ml heparin in saline (50 IU, #H3149, Sigma-Aldrich, USA) containing 0.33 mg/ml

gentamicin (#A506614, Sangon Biotech, Shanghai, China) before the morphine-SA training.

Morphine self-administration was carried out in mouse operant chamber (ENV-307W-CT, Med Associates, USA) located in an Expand PVC sound-attenuating cubicle (ENV-022V). The stainless-steel tubing of the catheter device was connected through a 25-gauge swivel (PHM-124B) and a variable speed syringe pump (PHM-100VS-2c). Data were recorded by a PC running MED-PC IV. Mice were trained to nose poke for morphine for 16 sessions (1 session/day). Each active nose-poke elicited the delivery of intravenous morphine hydrochloride (0.3 mg/kg/infusion, dissolved in 0.9% saline and delivered at 8.0 μL/s) and a tone-light cue (10 s, 2900 Hz tone with cue light above the nose poke while the house light was turned off). while the inactive nose poke had no consequences. Each session lasted for 2 hrs (0.3 mg/kg/inf, a maximum of 50 infusions). Mice were injected intraperitoneally with Mdivi-1 (50 mg/kg, in corn oil) or vehicle 45 min prior each session during the last 6 FR1 sessions. 1 day and 14 days after the last SA, cue-induced drug-seeking was tested for 60 min during which active nose pokes trigger the tone-light cue without drug infusion.

Quantification of accumulated faecal boli

To measure the morphine-induced constipation effects of Mdivi-1, the total accumulated faecal boli was assessed. Mice were injected with vehicle or Mdivi-1 (50 mg/kg and placed within a chamber before being placed on a mesh screen. Mice were maintained without food or water for 6 hrs. Faecal boli were collected in a box below the mesh and the cumulative mass was measured every hour for 6 hrs.

Single-cell RNA sequencing

Tissue dissection and neuronal ensembles isolation

TH-Cre mice infected with *AAV-RAM-TTA-TRE-Flex-tdTomato* were deeply anesthetized with isoflurane and then decapitated after ensembles labeling. Brains were quickly removed and transferred to cold choline solution (2.1 g/L $NaHCO_3$, 2.16 g/L glucose per, 0.172 g/L $NaH_2PO_4 \cdot H_2O$, 7.5 mM $MgCl_2 \cdot 6H_2O$, 2.5 mM KCl, 10 mM HEPES, 15.36 g/L choline chloride, 2.3 g/L ascorbic acid, and 0.34 g/L pyruvic acid) containing AP-5, TTX, actinomycin D and triptolide. The VTA was then micro dissected into 300-μm sections on a vibratome (Thermo Scientific, Inc.) and incubated on ice for 5 min in dissociation solution (HBSS containing 20 U/mL papain) were saturated with 95% O_2 and 5% CO_2. The tissue was dissociated for 1 h at 37 °C with gentle agitation. After centrifugation, the cells were washed and resuspended in dissociation solution containing 0.04% BSA and 15% Optiprep (Sigma). The tdTomato$^+$ cells were collected from VTA tissues of four mice in each group. The tdTomato$^+$ neuronal ensembles were manually picked up under a fluorescence microscope for scRNA-seq.

Library preparation and RNA sequencing

Single-cell transcriptome profiling was performed according to the Smart-Seq2 protocol. First, single cells were picked into 96-well plates which filled with 4 μL per well of lysis buffer containing 2.5 μM oligo-dT_{30}VN primer, 2.5 mM dNTP, 0.1% (v/v) Triton-X 100 and frozen at -86 °C until use. Reverse transcription was performed with template-switch oligo and HiScript II Reverse Transcriptase (Vazyme, Inc.), and then amplification using 2 × Phanta Max Master Mix (Vazyme, Inc.) as described for Smartseq2. Then the PCR products with purified, sonicated into ~300 bp fragments and subjected to library preparation (TruePrep DNA Library Prep Kit V2 for Illumina, Vazyme, Inc.). The libraries were processed on the Illumina platform for sequencing of 150 bp pair-end reds, generating approximatively 500 M of raw data were acquired for each cell. Single-cell RNA sequencing date have

been deposited in the Gene Expression Omnibus under accession number PRJNA949982.

Single-cell RNA-seq analysis

After initial quality check with FastQC, raw sequences were demultiplexed using zUMI software. Reads were aligned to the mouse genome GRCm38 genome using STAR (version 2.7.0a). Cells with a gene number of < 2000 were removed. And total 168 cells were collected for subsequent analysis (Sal-Ens group: 58 cells, Mor-Ens group: 54 cells, Mor-Ens with morphine-EDA group: 56 cells). DESeq2 was used to analyze differentially expressed genes (DEGs) analysis within three groups using a cutoff $|\log_2$Fold Change$|$ > 1 and p-value < 0.05. GO enrichment analysis was performed using ClusterProfiler.

Ribosome-associated transcripts

Purification of mRNA from neuronal ensembles with Ribo-tag

The purification procedure was modified and conducted as previously described (1, 2). *AAV-RAM-tTA-TRE-flpo*, fDIO-TH-Cre, and *AAV-FLEX-NBL10* were injected into the VTA of *wild-type* mice. Three weeks later, dopaminergic ensembles recruited by morphine or saline were labeled. 12 hrs, 1 d, and 6 d after chronic morphine administration, the brain sections containing the VTA were rapidly dissected and immediately homogenized in ice-cold supplemented hybridization buffer containing: 25 mM Tris [pH = 7.0], 25 mM Tris [pH = 8.0], 12 mM $MgCl_2$, 100 mM KCl, 1% Triton X-100, 1 mM DTT (646563-10X, Sigma), 1×protease inhibitors (04693159001, Roche), 200 units/mL RNase inhibitor (N2112S, Promega), 100 μg/mL cycloheximide (14126, Cayman), 1 mg/ml heparin (H3393, Sigma-Aldrich). Homogenates were centrifuged at 10,000 rpm, 4°C for 10 min. The remaining supernatant lysate (output) was incubated with 10 μg rabbit anti-HA antibody (H6908, Sigma-Aldrich) and 100 μL

Dynabeads Protein G (10003D, Novex) rotated overnight at 4°C. Beads were collected on a magnetic rack, and washed three times with high-salt polysome wash buffer. Purified mRNA was eluted from the Dynabeads after DNase digestion using SuPerfectRI Total RNA Isolation Reagent (R401-01, Novex) according to the manufacturer's instructions. Agilent RNA 6000 Pico Kit (5067-1513, Agilent) and the Agilent 2100 Bioanalyzer were used to evaluate the quality and concentration of the purified mRNA.

Reverse transcription and qPCR. Reverse transcription was completed using the PrimeScript RT Reagent Kit (RR037A, Takara Biotechnology, Dalian, China). The cDNA was subjected to qPCR using SYBR Premix Ex Taq (RR420A, Takara) and Eppendorf Mastercycler PCR System (Eppendorf, Hamburg, Germany). The primers synthesized by Genewiz (Azenta, Suzhou, Jiangsu, China**)** were listed in **Supplementary Table 1**.

Ex vivo electrophysiology recording

Coronal slices (300 μm thick) containing the VTA were prepared as previously described (2, 3). Briefly, the brains were rapidly removed and the coronal slices (300 μm) containing the VTA were prepared using a vibratome (HM650V, Thermo Scientific, Wilmington, MA, USA) and kept at room temperature in modified artificial cerebrospinal fluid (ACSF) (in mM: 94 NaCl, 2.5 KCl, 1.2 NaH_2PO_4, 30 $NaHCO_3$, 20 HEPES, 25 glucose, 5 sodium ascorbate, 2 thiourea, 3 sodium pyruvate, 1.2 $MgCl_2$ and 2.4 $CaCl_2$). For whole-cell patch clamp recording, the slices were kept at 32 -34°C and perfused with oxygenated recording ACSF (in mM: 124 NaCl, 2.5 KCl, 1.2 NaH_2PO_4, 24 $NaHCO_3$, 5 HEPES, 12.5 glucose, 1.2 $MgCl_2$ and 2.4 $CaCl_2$) at a rate of 3 mL/min. $tdTomato^+$ neurons were identified under a

fluorescence microscope (Olympus BX51WI, Tokyo, Japan). The EPC-10 amplifier and Patchmaster software (HEKA Elektronik, Lambrecht/Pfalz, Germany) were used to measure holding and synaptic responses. The pipette resistance was in the range of 5-8 MΩ. Data were filtered at 2.9 kHz and sampled at 10 kHz. Recordings with R_s > 30 MΩ were excluded from statistical analysis. Data were analyzed off-line with Clampfit 10.3 (Molecular Devices, Union City, CA, USA) or Mini Analysis Program (Synaptosoft Inc, Fort Lee, NJ, USA).

For action potential (AP) recording, spike fidelity was measured by the reliability of eliciting an action potential in response to somatically injected currents. K^+-based intracellular solution (in mM: 130 K-gluconate, 6 KCl, 2 $MgCl_2$, 10 HEPES, 2.5 ATP-Mg, 0.5 GTP-Na_2, 10 creatine phosphate, 0.6 EGTA, 0.5% biocytin, pH = 7.25, 290 mOsm) was used for AP recording (2). A current-step protocol (from -50 to +150 pA, with a 10-pA increment) lasting 1 s was performed and repeated. After the recording of a particular cell, the number of evoked APs was compared across all runs; cells with a run-up or run-down >15% were excluded. Rheobase was measured by injecting a variable positive current step (2 pA increment from the start of 0 pA) lasting 800 ms until the cell discharged a single AP. Membrane input resistance was measured by injecting a negative current step of 100 pA for 1s while holding the membrane potential to -70 mV. The after-hyperpolarization potential (AHP) was sampled after the first AP spike, usually elicited by the rheobase current step. The AP threshold was measured at the point of inflection when the slope exceeded 5 mV/ms. Neurons were held at 0 pA under a current-clamp mode to record spontaneous firing for 3 min, and the neurons with regular spontaneous firing were used for firing frequency analysis (4, 5).

To record evoked IPSCs or EPSCs recording, neurons were held at -70 mV to record evoked EPSCs and at 0 mV to record evoked IPSCs under voltage-clamp mode from the same dopaminergic neurons.

A stainless steel concentric bipolar stimulating electrode (FHC) was placed about 100-200 μm rostral to the recording electrode. A modified intracellular solution (127.5 mM cesium methanesulfonate, 7.5 mM CsCl, 10 mM HEPES, 2.5 mM $MgCl_2$, 4 mM Na_2ATP, 0.4 mM Na_3GTP, 10 mM sodium phosphocreatine, 0.6 mM EGTA and 0.4% biocytin, pH 7.25, 290 mOsm) was used to adjust the reversal potential of the γ-aminobutyric acid-A receptor (GABAAR) response. The AMPA EPSC and GABA IPSC were isolated at different holding potential after bath application of NMDA antagonist D-APV. The EPSC/IPSC ratio was calculated by dividing the peak amplitudes. Stimulation artifacts were removed from the presentative traces.

Single-molecule RNA Fluorescence in Situ Hybridization (smFISH)

As previously described (2, 3), mice were anesthetized with isoflurane and transcardially perfused with 0.9% saline followed by 4.0 % paraformaldehyde in 0.1 M Na_2HPO_4/NaH_2PO_4 buffer (pH = 7.4). After post-fixation and dehydration, 10-μm thick frozen brain slices were coronally sectioned with Cryostat (CM3050 S, Leica, Buffalo Grove, IL, USA) and mounted onto Superfrost Plus microscope slides (Fisher Scientific, USA). smFISH was performed according to the RNAscope procedures (Advanced Cell Diagnostics, ACD, USA). Sections were incubated with the probes against mouse *Mfn1* (#581881-C3), *tdTomato* (#317041-C2), and *Egfp* (#400281-C2) for 2 hrs at 40℃. After hybridization, signals were amplified using the RNAscope® Multiplex Fluorescent Detection Kit v2 (#323110, ACD) were used to amplify signals. Images were acquired using a Nikon A1 microscope with 20× objective and analyzed by the investigators blinded to group assignment. IOD in EGFP-positive neurons were analyzed by Image-Pro Plus 6.0 (Media Cybernetics, Rockville, MD, USA).

Immunohistochemistry

Mice were anesthetized with isoflurane and perfused with 4% paraformaldehyde (PFA, dissolved in 0.1 M PBS). Brains were removed, fixed in 4% PBS-buffered PFA overnight and subjected to dehydration in graded sucrose solutions (20%–30%) at 4°C. The frozen coronal slices of 30 μm thicknesses were sectioned at 30 μm by Cryostat. Brain sections were washed in PBS and then incubated in primary antibody in blocking buffer (10 % normal goat serum and 0.2% Triton-X-100) over light. Then slices were washed and incubated in secondary antibodies for 1 hr and DAPI for 5 min at room temperature. After washing in PBS, brain slices were mounted with anti-quenching mounting medium. Primary antibodies used: anti-HA (H6908-0.5 ml, Sigma, 1:500), anti-TH antibodies (MAB318, Merck, 1:1000), anti-nitrotyrosine (sc-32757, Santa Cruz, 1:200), anti-COXIV (11242-1-AP, Proteintech, 1:200), and DAPI (D9534, Sigma, 1:10000). Fluorescent secondary antibodies used were: anti-mouse 488 (711-545-150, Jackson ImmunoResearch, 1:1000) and anti-rabbit Cy3 (115-165-116, Jackson ImmunoResearch, 1:500). Images were acquired using a confocal microscope (Nikon-1A, Japan) with a 20× air objective.

Reference

1. He G, Huai Z, Jiang C, Huang B, Tian Z, Le Q, et al. Persistent increase of accumbens cocaine ensemble excitability induced by IRK downregulation after withdrawal mediates the incubation of cocaine craving. *Molecular psychiatry.* 2023;28(1):448-62.
2. Jiang C, Wang X, Le Q, Liu P, Liu C, Wang Z, et al. Morphine coordinates SST and PV interneurons in the prelimbic cortex to disinhibit pyramidal neurons and enhance reward. *Molecular psychiatry.* 2021;26(4):1178-93.
3. Jiang C, Yang X, He G, Wang F, Wang Z, Xu W, et al. CRH(CeA-->VTA) inputs inhibit the positive ensembles to induce negative effect of opiate withdrawal. *Molecular psychiatry.* 2021;26(11):6170-86.
4. Frau R, Miczan V, Traccis F, Aroni S, Pongor CI, Saba P, et al. Prenatal THC exposure produces a hyperdopaminergic phenotype rescued by pregnenolone. *Nat Neurosci.* 2019;22(12):1975-85.
5. Chen M, Zhao Y, Yang H, Luan W, Song J, Cui D, et al. Morphine disinhibits glutamatergic input to VTA dopamine neurons and promotes dopamine neuron excitation. *Elife.* 2015;4.

SUPPLEMENTARY FIGURES

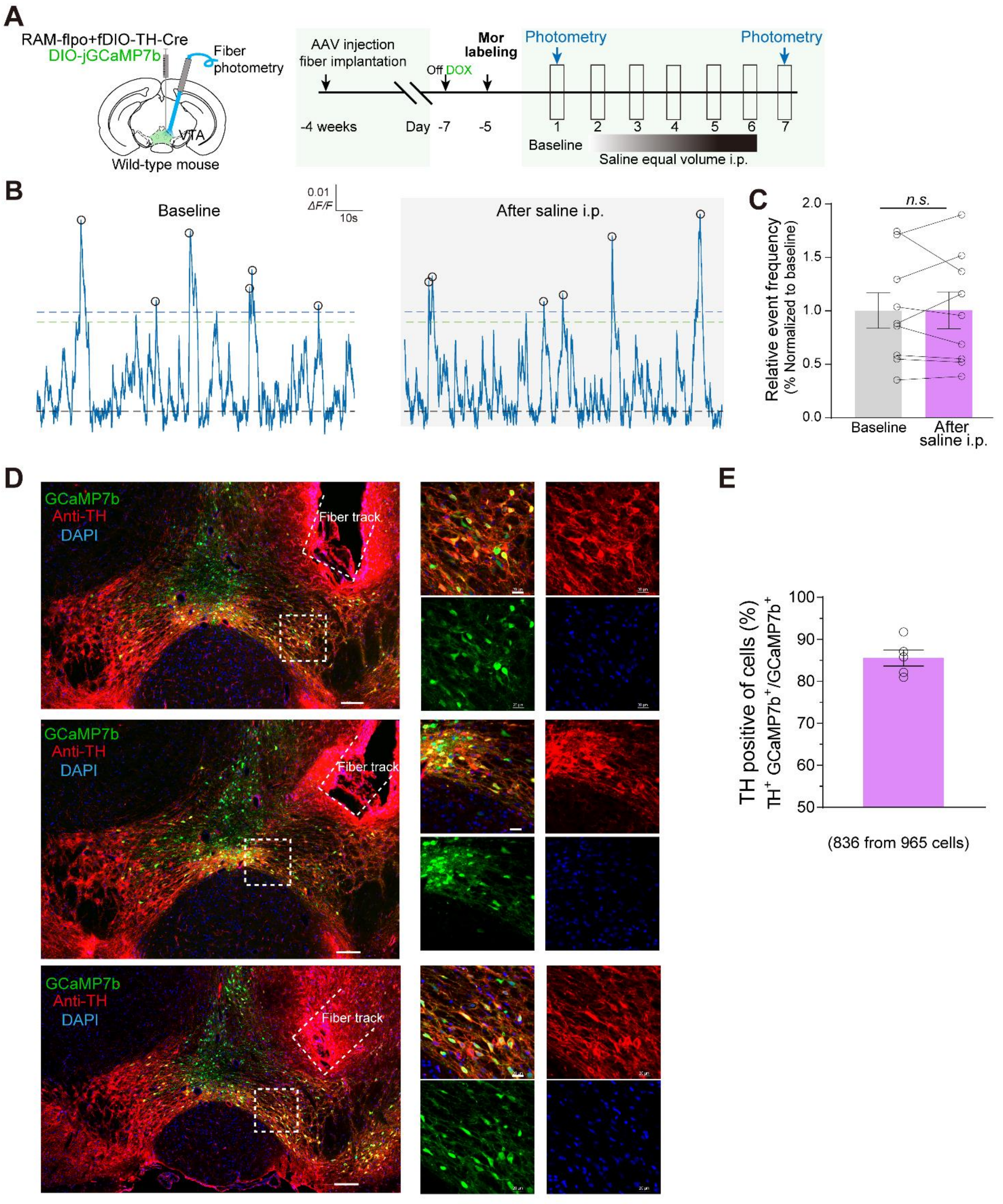

Figure S1. Ca^{2+} events of VTA dopaminergic Mor-Ens in the mice from saline control group. Related to Figure 1.

(**A**) Schematic of virus injection and fiber photometry recordings. Viruses combining Cre-loxp and Flp-FRT systems were used to label TH^+ neuronal ensembles with GCaMP7b, and the optic fiber was unilaterally implanted in the VTA of mice. (**B**) Representative GCaMP7b photometry traces from Mor-Ens before and after saline treatment. Circles above trace indicate threshold-detected events. (**C**) Relative frequency of calcium events in Mor-Ens. Paired t-test, $n = 9$ mice. (**D**) Representative images of TH staining in the VTA. Green: GCaMP7b; Red: TH; Blue: DAPI. Scale bars, 100 μm, 20 μm. (**E**) Quantification of the TH^+ components in Mor-Ens. n = 965 cells from 5 mice. Data are presented as mean ± S.E.M; *n.s.* not significant.

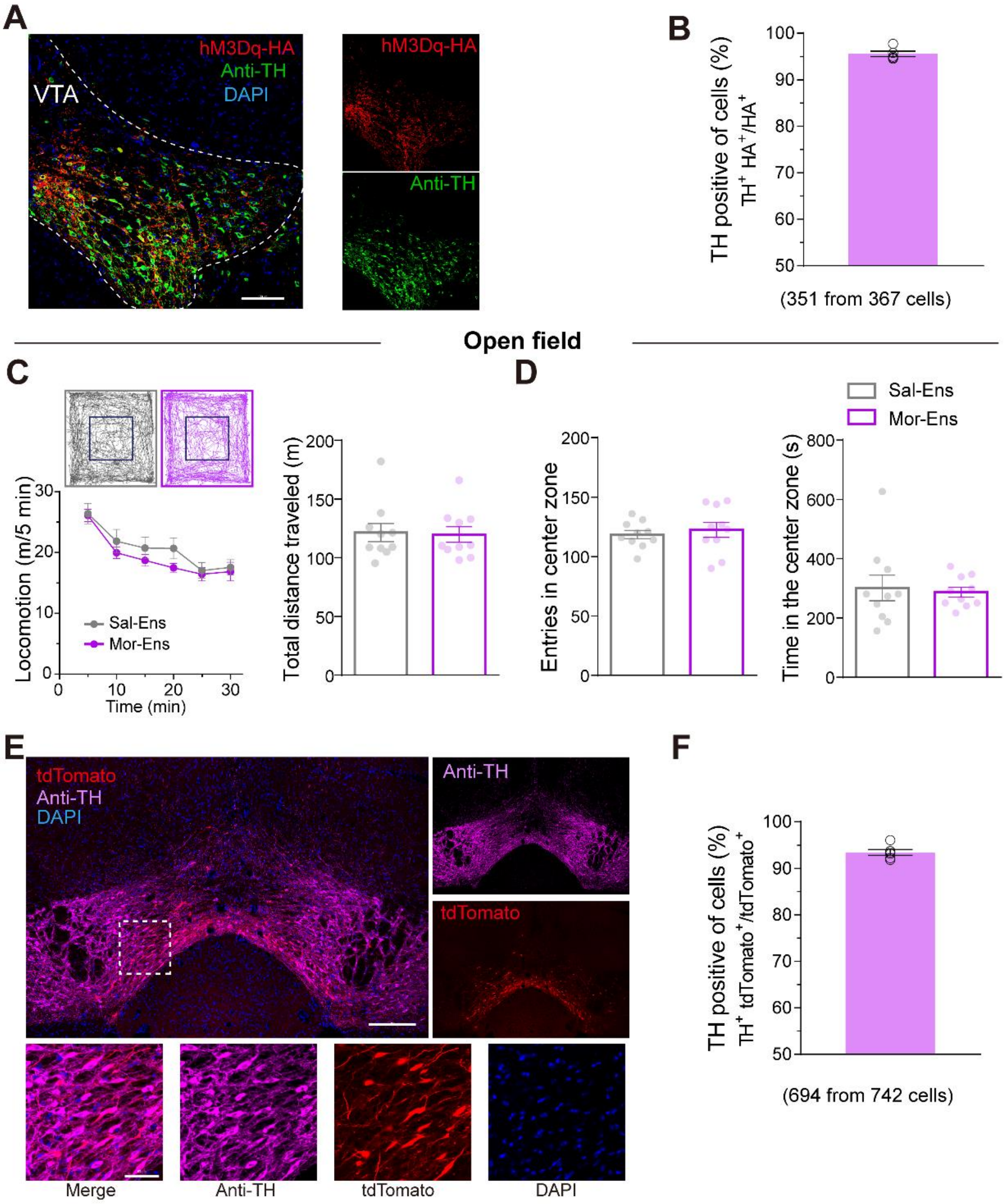

Figure S2. Verification of labeling approach in *TH-Cre* mice and the effects of chemogenetic activating VTA dopaminergic Mor-Ens on locomotor activity during morphine withdrawal. Related to Figure 1 or 2.

(**A**) Representative images of TH staining in the VTA. Mor-Ens expressing in the VTA of *TH-Cre* mouse. Red: hM3Dq-HA; Green: TH; Blue: DAPI. Scale bar, 100 μm. (**B**) Quantification of the TH^+ components in $hM3Dq^+$ Mor-Ens. n = 367 cells from 5 mice. (**C**) Representative video-tracked path

and average locomotion within 5 min (left) and total distance traveled (right). 10 mice/group, two-way RM ANOVA for average locomotion, Unpaired t-test for total distance. (**D**) Entries and time in the center zone of the open field within 30 min. 10 mice/group, Unpaired t-test. (**E**) Representative images of TH staining in the VTA. Red: tdTomato; Magenta: TH; Blue: DAPI. Scale bars, 100 μm, 50 μm. (**F**) Quantification of the TH^+ components in $tdTomato^+$ Mor-Ens. n = 742 cells from 6 mice. Data are presented as mean ± S.E.M; *n.s.* not significant.

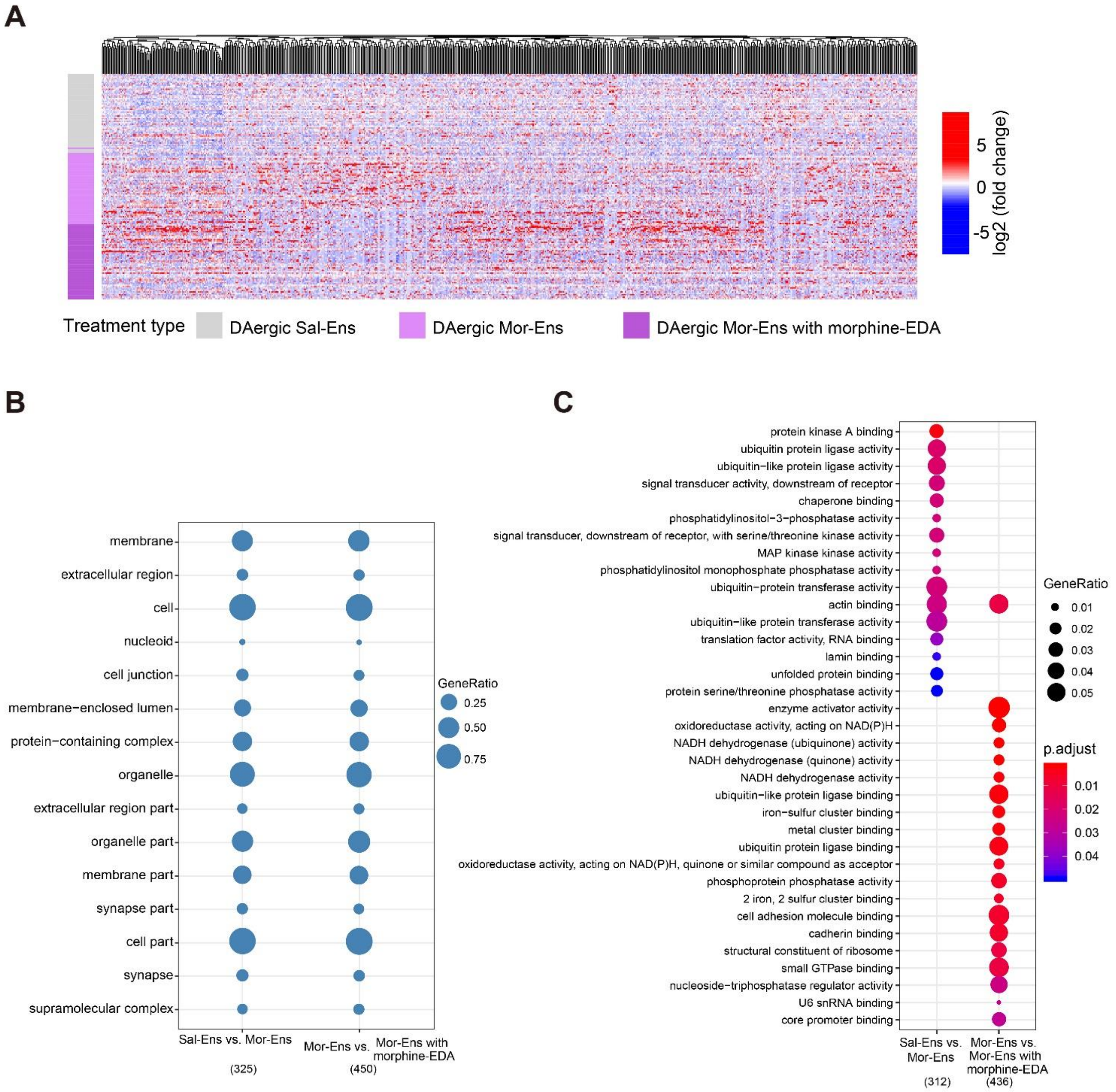

Figure S3. Analysis of the differential expression genes among Sal-Ens, Mor-Ens, and Mor-Ens treated with morphine EDA. Related to Figure 2.

(**A**) Heatmap of the differentially expressed genes among the dopaminergic Sal-Ens, Mor-Ens, and Mor-Ens treated with escalating dose morphine administration (morphine EDA). The differentially expressed genes are ordered by hierarchical clustering of the expression difference. (**B** and **C**) Pathway enrichment analysis of the differentially expressed genes between Sal-Ens *vs* Mor-Ens, and between Mor-Ens without *vs* with morphine EDA. The differentially expressed genes enriched in pathways

with commonalities (**B**) and differences (**C**).

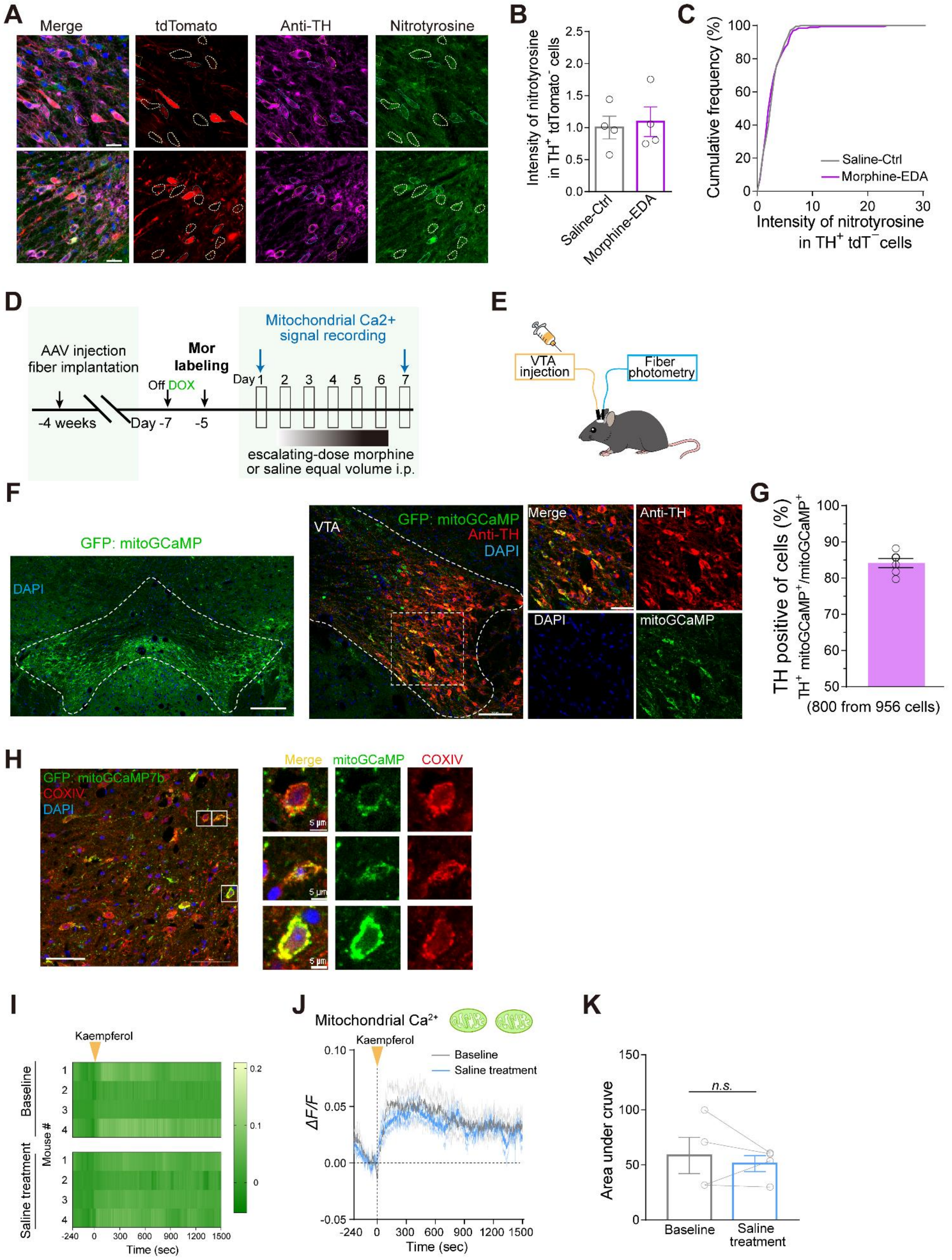

Figure S4. Quantification of the nitrotyrosine levels in non-labelled dopaminergic neurons and

mitochondrial Ca^{2+} dynamics of VTA dopaminergic Mor-Ens. Related to Figure 3.

(**A**) Representative images of nitrotyrosine staining of the brain section containing VTA from mice with or without morphine-EDA. Dashed white lines outlining non-labelled DA neurons. Red: tdTomato; Magenta: TH; Green: nitrotyrosine; Blue: DAPI. Scale bar: 20 μm. (**B**) Normalized florescence intensity of nitrotyrosine in VTA TH^{+} $tdTomato^{-}$ neurons from saline and morphine-EDA groups. Unpaired t-test, *n* = 4 mice/group. (**C**) Cumulative frequency distribution of nitrotyrosine intensity in TH^{+} $tdTomato^{-}$ neurons. Two-sample Kolmogorov-Smirnov test, Saline-Ctrl: 210 cells; Morphine-EDA: 193 cells. (**D**) Experimental scheme of detecting mitochondrial Ca^{2+} signal in the VTA Mor-Ens in freely moving mice before or after morphine EDA. (**E**) Schematic of fiber photometry recording in Mor-Ens from the mice injected with kaempferol in the VTA (1.6 μL, 2 nmol/μL). (**F**) Representative images of Flpo- and Cre-dependent mito-GCaMP expression in the VTA Mor-Ens (left). Scale bar, 200 μm. Representative images of TH staining in the VTA (right). Green: mito-GCaMP; Red: TH; Blue: DAPI. Scale bar, 100 μm, 20 μm. (**G**) Quantification of the TH^{+} components in the $tdTomato^{+}$ Mor-Ens. n = 956 cells from 6 mice. (**H**) Representative images of COXIV co-localization with mito-GCaMP in the VTA. Red: COXIV; Green: mito-GCaMP7b; Blue: DAPI. Scale bars, 200 μm (left), 5 μm (right). (**I**) Heatmap of mito-GCaMP fluorescence in response to kaempferol in Mor-Ens with saline treatment. (**J**) Average $\Delta F/F$ (%) and (**K**) AUC quantification of mito-GCaMP fluorescence. Dashed vertical line marks the injection of kaempferol in the VTA. Paired t-test, *n* = 4 mice. Data are presented as mean ± S.E.M; *n.s.* not significant.

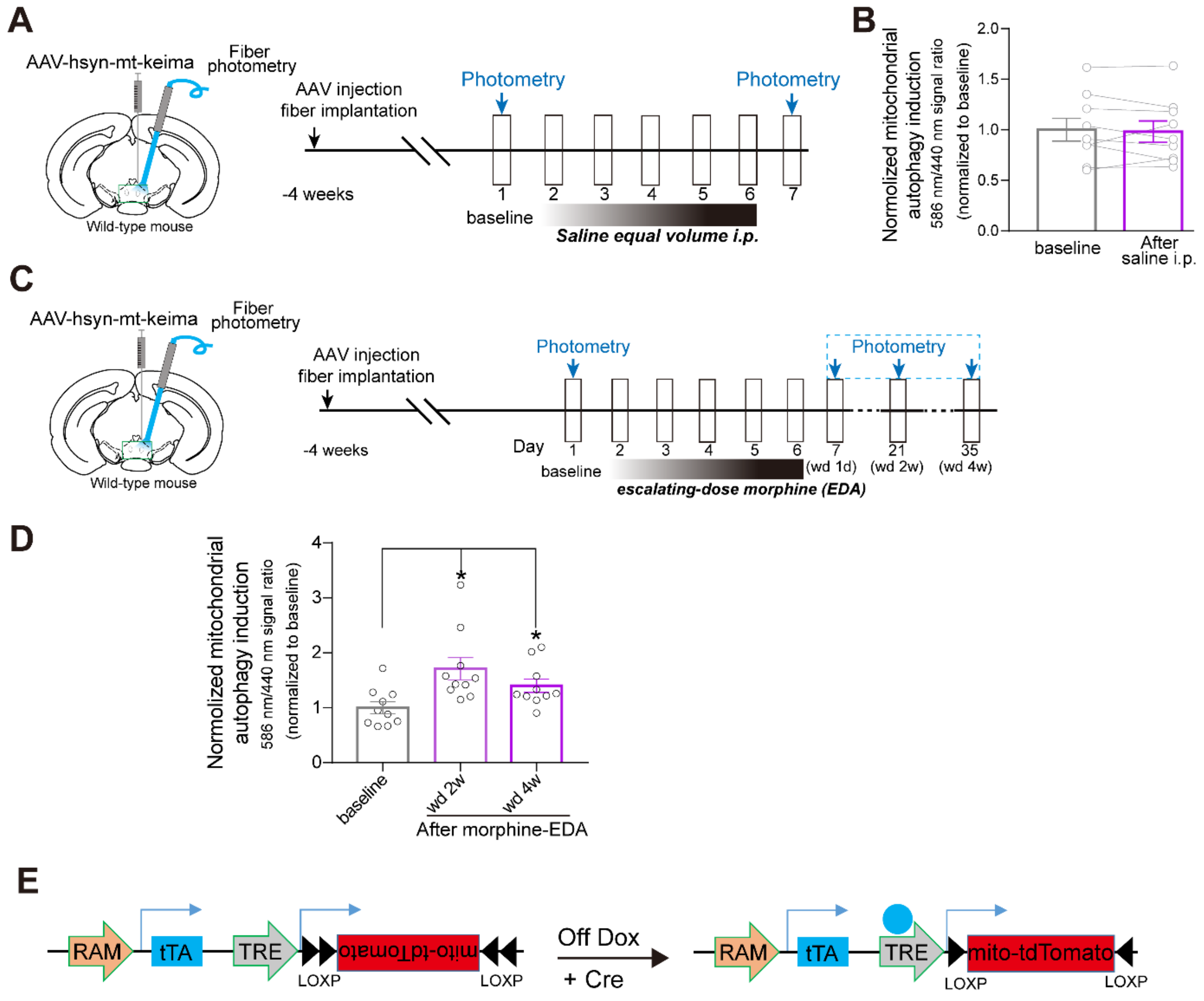

Figure S5. Increased mitophagy in VTA neurons after chronic morphine administration. Related to Figure 4.

(**A**) Experimental scheme of the three-color fiber photometry recording setup for detecting mitophagy in VTA neurons from the mice in saline control group. (**B**) Relative mitochondrial autophagy induction (% normalized to baseline) in the VTA after saline treatment. Paired t-test, n = 9. (**C**) Experimental scheme of the three-color fiber photometry recording setup for detecting mitophagy in VTA neurons at different time points after morphine EDA. (**D**) Relative mitochondrial autophagy induction (% normalized to baseline) in VTA at 2 w and 4 w after morphine EDA. One-way RM ANOVA, n = 10/group. (**E**) Schematic of Cre- and RAM-dependent AAV constructs for expressing mito-tdTomato in Mor-Ens. Data are presented as mean ± S.E.M; *n.s.* not significant, *P < 0.05.

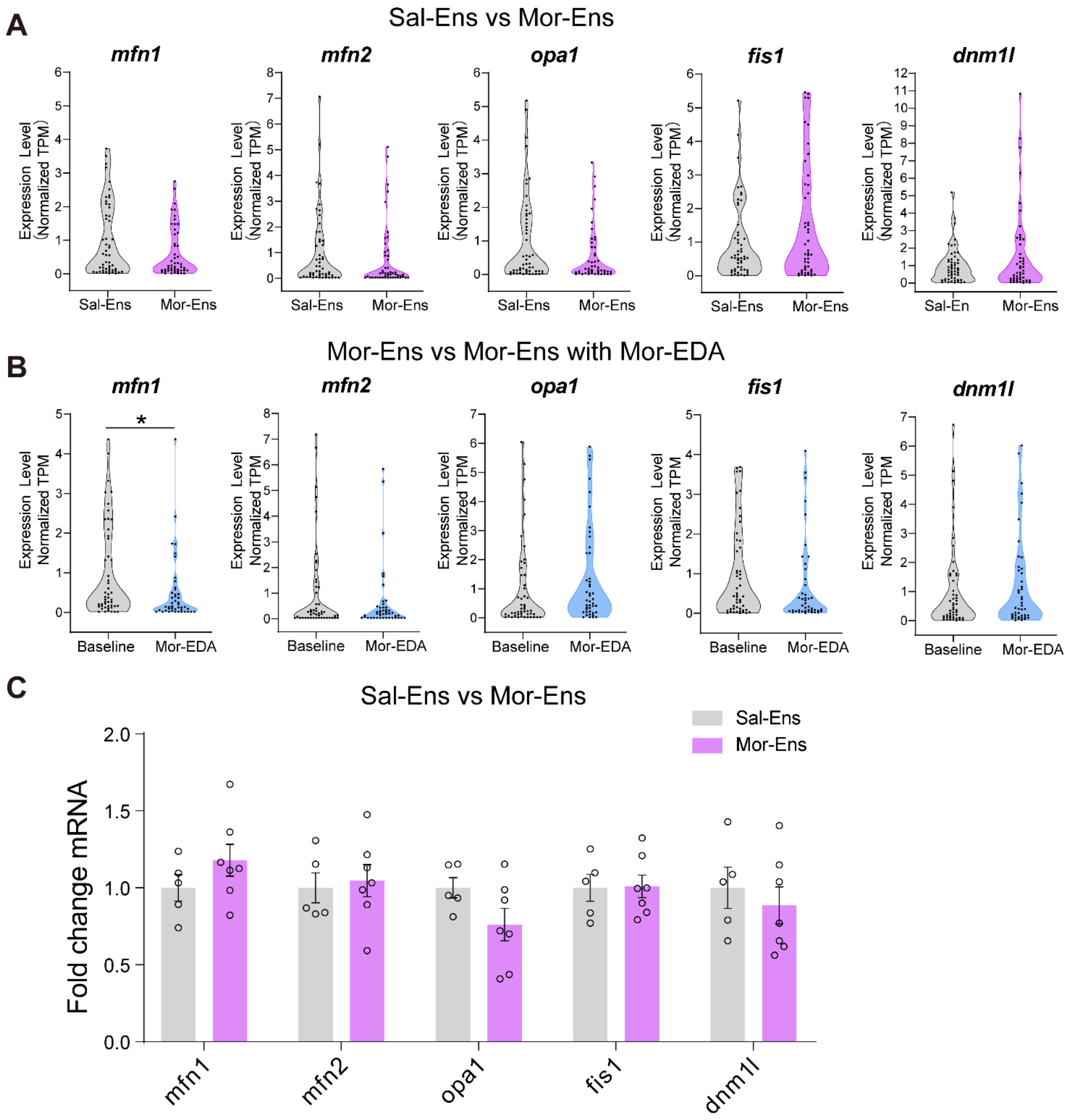

Figure S6. Expression of mitochondrial fission and fusion genes in VTA neuronal ensembles from single-cell sequencing and ribo-tag experiments. Related to Figure 5.

(**A** and **B**) Analysis of relative transcription levels of fission (*dnm1l*, *fis1*) and fusion (*mfn1*, *mfn2*, and *opa1*) genes in VTA dopaminergic Sal-Ens and Mor-Ens, Sal-Ens *vs* Mor-Ens, Mor-Ens without *vs* with Mor-EDA. Sal-Ens: 52 cells from 4 mice; Mor-Ens: 49 cells from 4 mice; Mor-Ens with Mor-EDA: 44 cells from 4 mice, unpaired t-test or Mann-Whitney test for 2 groups. (**C**) Quantification of

the ribosome-associated mRNA levels of fission (*dnm1l*, *fis1*) and fusion (*mfn1*, *mfn2*, and *opa1*) genes in the VTA dopaminergic ensembles. Sal-Ens vs Mor-Ens, 5-7 mice/group, Unpaired t-test. Data are presented as mean ± S.E.M. *$P < 0.01$.

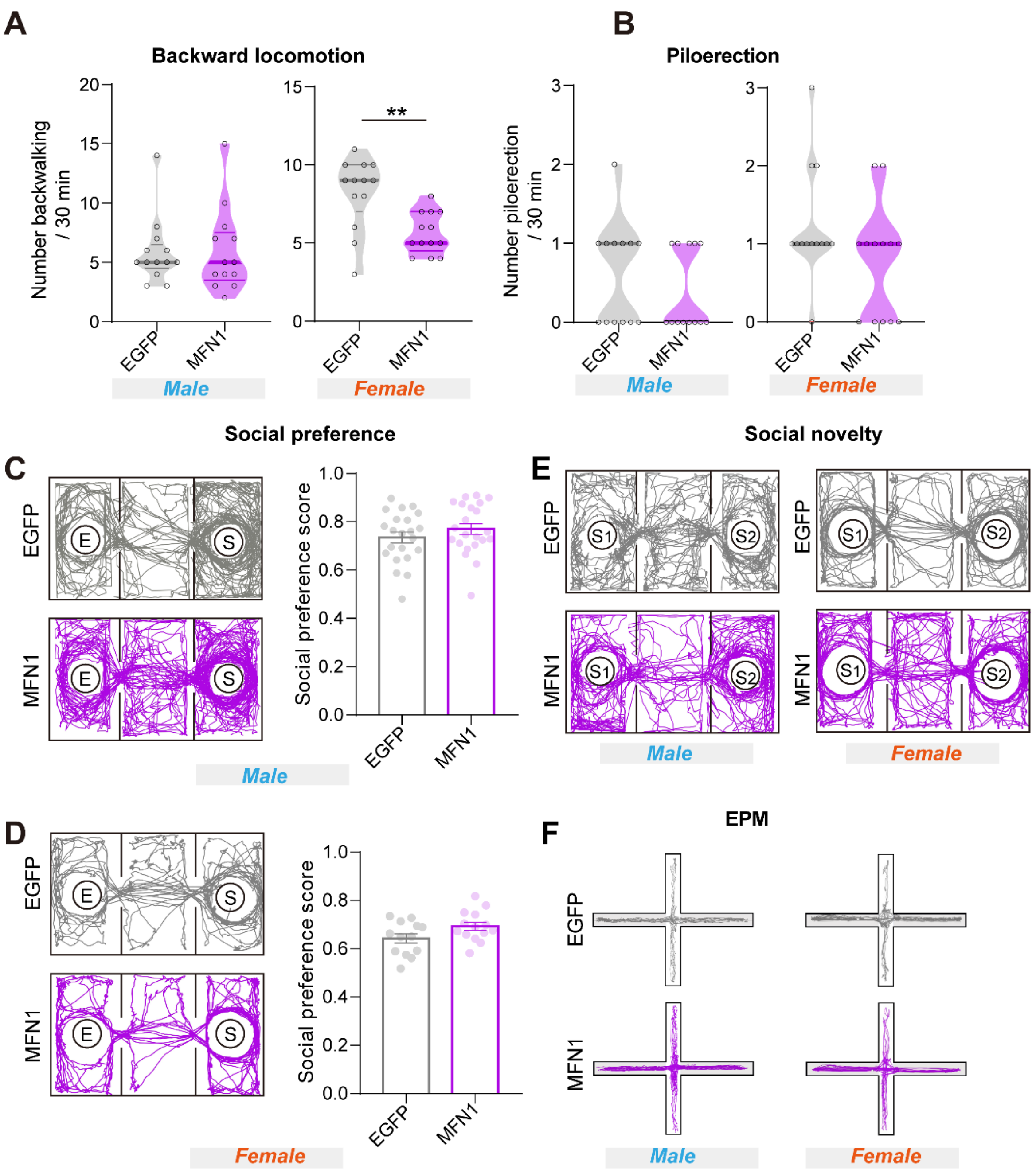

Figure S7. The effect of overexpressing MFN1 in the VTA dopaminergic Mor-Ens on behavioral performance during morphine withdrawal in both male and female mice. Related to Figure 6 or 7.

(**A** and **B**) The effect of overexpressing MFN1 in the VTA dopaminergic Mor-Ens on naloxone-precipitated withdrawal symptoms in both male and female mice. Backward locomotion (**A**) and piloerection (**B**) were analyzed from the mice in EGFP control or MFN1-EGFP groups. Male: 12-13

mice/group; female: 13 mice/group. (**C-F**) The effect of overexpressing MFN1 in the VTA dopaminergic Mor-Ens on negative affect during spontaneous morphine withdrawal in both male and female mice. Representative tracks of social preference (**C** and **D**), social novelty (**E**) and EPM (**F**) were shown. Social preference score was analyzed from the mice in EGFP control or MFN1-EGFP groups (**C** and **D**). Male: 11-22 mice/group; female: 12-14 mice/group. Gender-segregated unpaired t-test or Mann-Whitney test between EGFP and MFN1-EGFP groups. Data are presented as mean ± S.E.M; $^{**}P < 0.01$.

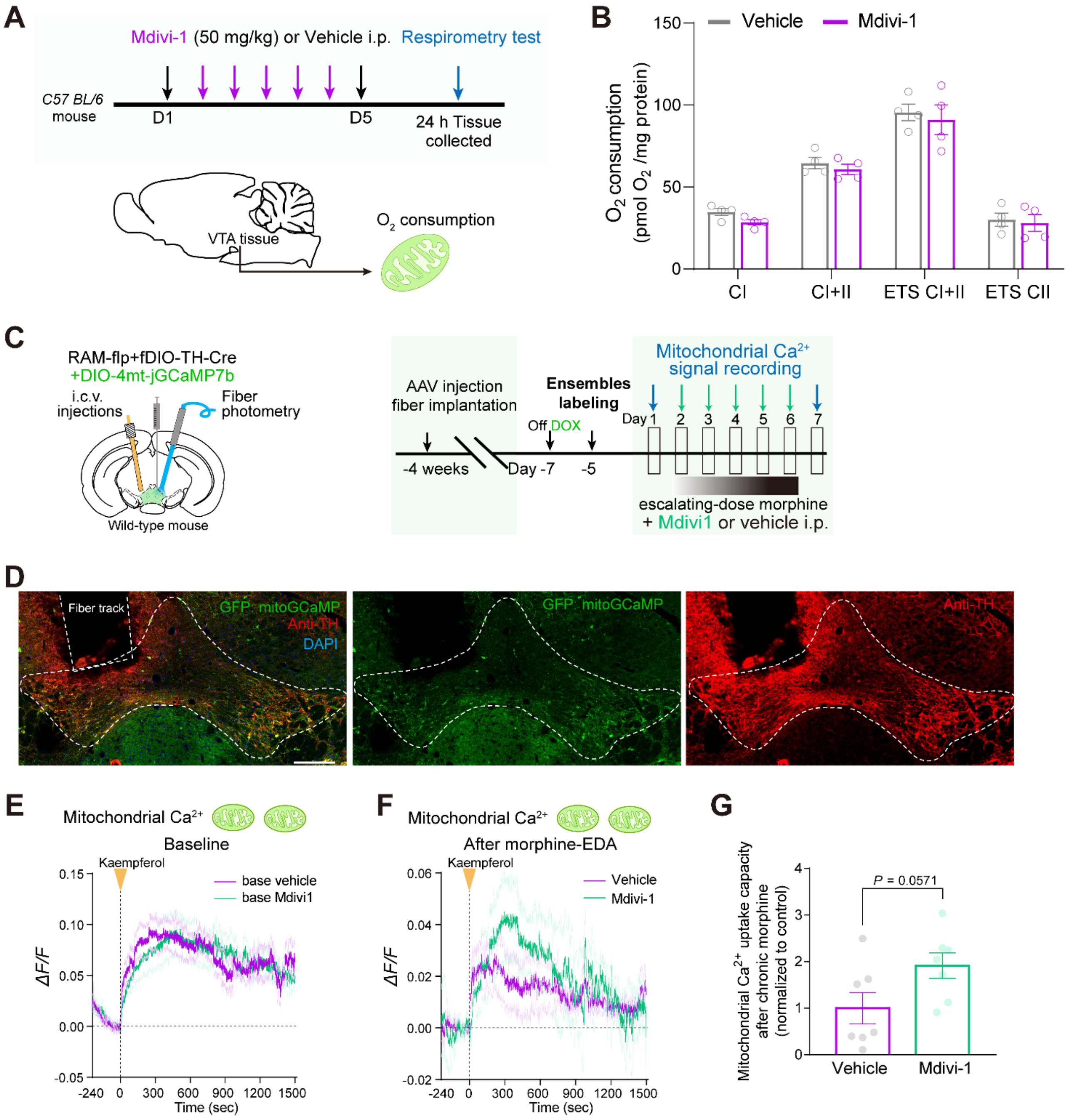

Figure S8. The effect of Mdivi-1 treatment on the VTA mitochondrial respiration of mice without morphine EDA, and on the mitochondrial Ca^{2+} dynamics in the VTA dopaminergic Mor-Ens of mice from saline and morphine EDA groups. Related to Figure 8.

(**A**) Experimental scheme for assessing the mitochondrial respiration of the VTA in vehicle and Mdivi-1 (50 mg/kg, i.p.) treatment groups. (**B**) Oxygen consumption rate of mitochondria in the VTA from vehicle and Mdivi-1 groups. 4 mice/group, two-way RM ANOVA by Bonferroni's test. (**C**)

Experimental scheme of fiber photometry setup for detecting mitochondrial Ca^{2+} signal in the VTA dopaminergic Mor-Ens following morphine EDA in freely moving mice. (**D**) Representative images showing the expression of mito-GCaMP and TH-staining in the VTA. Dashed white lines outline the VTA and the fiber optic tract. Green: mito-GCaMP; Red: TH; Blue: DAPI. Scale bar, 200 μm. (**E** and **F**) Average *ΔF/F* (%) mito-GCaMP fluorescence in Mor-Ens in response to kaempferol (1.6 μL, 2 nmol/μL in the VTA) in vehicle and Mdivi-1 groups before (**E**) and after morphine-EDA (**F**). Dashed vertical line indicates the injection of kaempferol. (**G**) AUC quantification of mito-GCaMP fluorescence. Unpaired t-test, n = 7/groups. Data are presented as mean ± S.E.M.

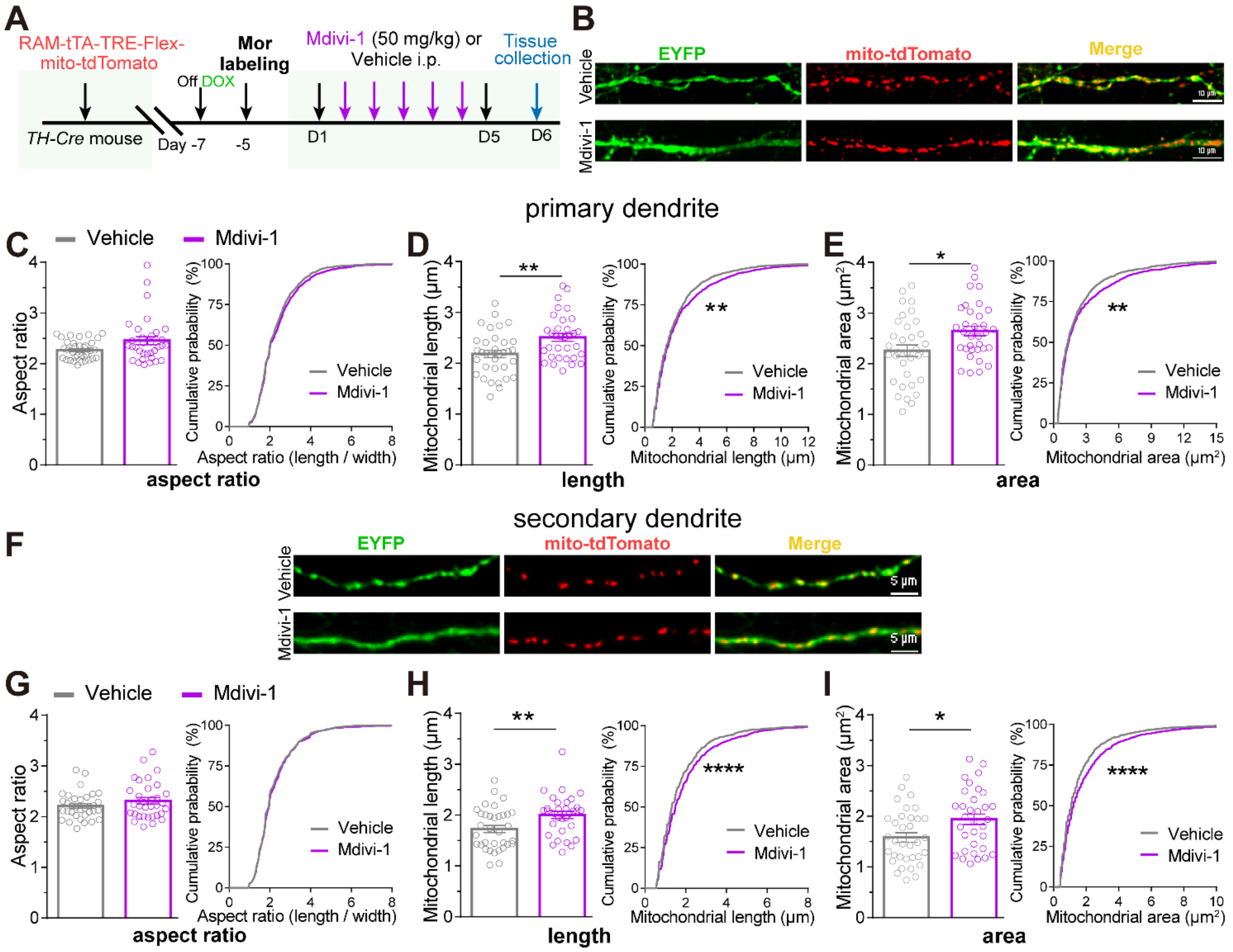

Figure S9. The effect of Mdivi-1 treatment on mitochondrial morphology of VTA dopaminergic Mor-Ens from the mice without morphine EDA. Related to Figure 8.

(**A**) Experimental scheme for tracing mitochondrial morphology in dopaminergic Mor-Ens from vehicle and Mdivi-1 groups without chronic morphine administration. (**B**) Representative images of primary dendrites (green) containing labeled mitochondria (red) in Mdivi-1 or vehicle groups. Red: mito-tdTomato; Green: EYFP. Scale bar: 10 μm. (**C-E**) Quantification of mitochondrial aspect ratio (**C**), length (**D**)and area (**E**) in primary dendrites of dopaminergic Mor-Ens in vehicle (34 neurons/5 mice) and Mdivi-1 groups (33 neurons/4 mice). Unpaired t-test and Kolmogorov-Smirnov test. (**F**) Representative images of secondary dendrites (green) containing labeled mitochondria (red) in Mdivi-1 or vehicle groups. Red: mito-tdTomato; Green: EYFP. Scale bar: 5 μm. (**G-I**) Quantification of

mitochondrial aspect ratio (**G**), length (**H**)and area (**I**) in secondary dendrites of the dopaminergic Mor-Ens in vehicle (34 neurons/5 mice) and Mdivi-1 groups (33 neurons/4 mice). Unpaired t-test and Kolmogorov-Smirnov test. Data are presented as mean ± S.E.M; *P < 0.05, **P < 0.01, ****P < 0.0001.

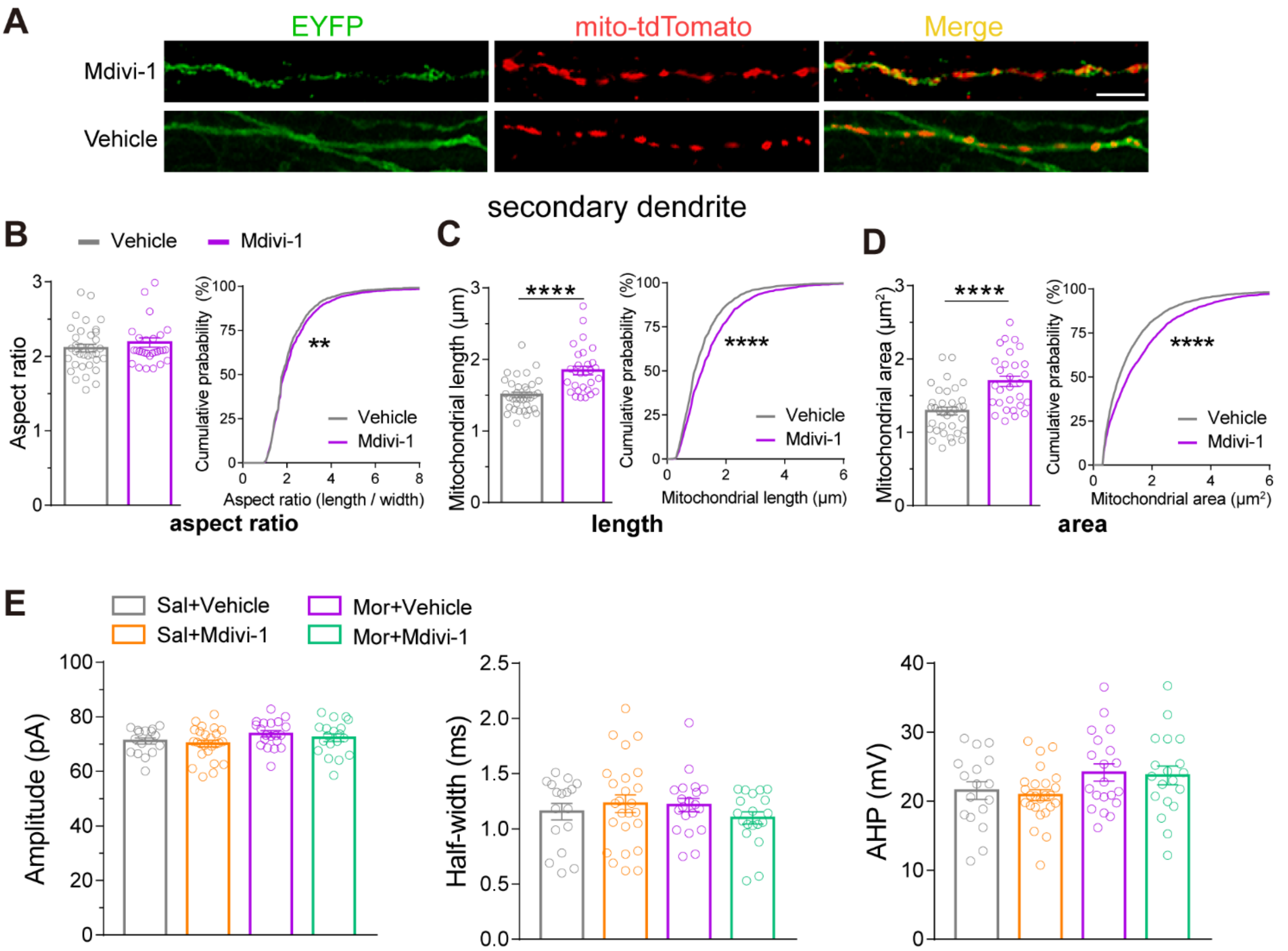

Figure S10. The effect of Mdivi-1 on the dendritic mitochondrial morphology and the electrophysiological properties of dopaminergic Mor-Ens. Related to Figure 9.

(**A**) Representative images of secondary dendrites of the dopaminergic Mor-Ens from the mice treated with Mdivi-1 or vehicle during escalating dose morphine administration. Green: EYFP; Red: mito-tdTomato. Scale bar: 5 μm. (**B-D**) Quantification of mitochondrial aspect ratio (**B**), length (**C**)and area (**D**) in secondary dendrites of dopaminergic Mor-Ens from vehicle (35 neurons/6 mice) and Mdivi-1 (29 neurons/8 mice) groups. Kolmogorov-Smirnov test, Unpaired t-test or Mann-Whitney test for 2 groups. (**E**) Quantification of the amplitude, half-width and AHP of the dopaminergic Mor-Ens in the VTA. 17-25 neurons from 4-5 mice/group, two-way ANOVA with Bonferroni's test in (**E**). Data are presented as mean ± S.E.M; $**P < 0.01$, $****P < 0.0001$.

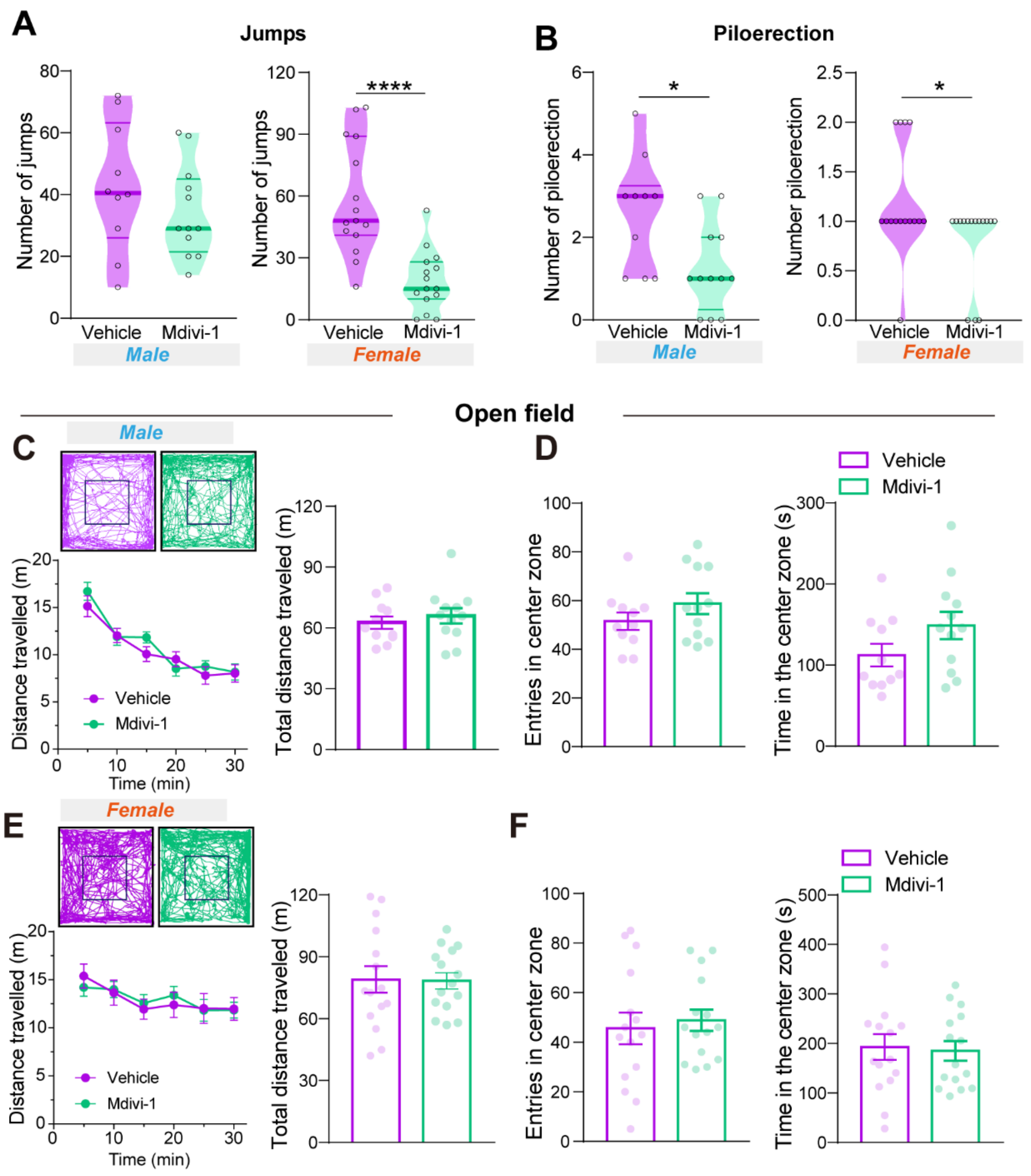

Figure S11. The effect of Mdivi-1 on naloxone-precipitate withdrawal symptoms and the open-field test during spontaneous morphine withdrawal in both male and female mice. Related to Figure 10.

(**A** and **B**) The effect of Mdivi-1 on jumps (**A**) and piloerection (**B**) induced by naloxone-precipitated withdrawal were analyzed in vehicle and Mdivi-1 pre-treated groups. Male: 10-12 mice/group; female: 15 mice/group, unpaired t-test or Mann-Whitney test. (**C and F**) Representative track, average locomotion with 5 min, and total distance traveled in the open field of male (**C**) and female (**F**) mice.

(**D and F**) Entries and time in the center zone in the open field within 30 min in male (**D**) and female (**F**) mice. Male: 11-12 mice/group; female: 14-15 mice/group. Two-way RM ANOVA for average locomotion, unpaired t-test for two groups. Data are presented as mean ± S.E.M; *$P < 0.05$, ****$P < 0.0001$.

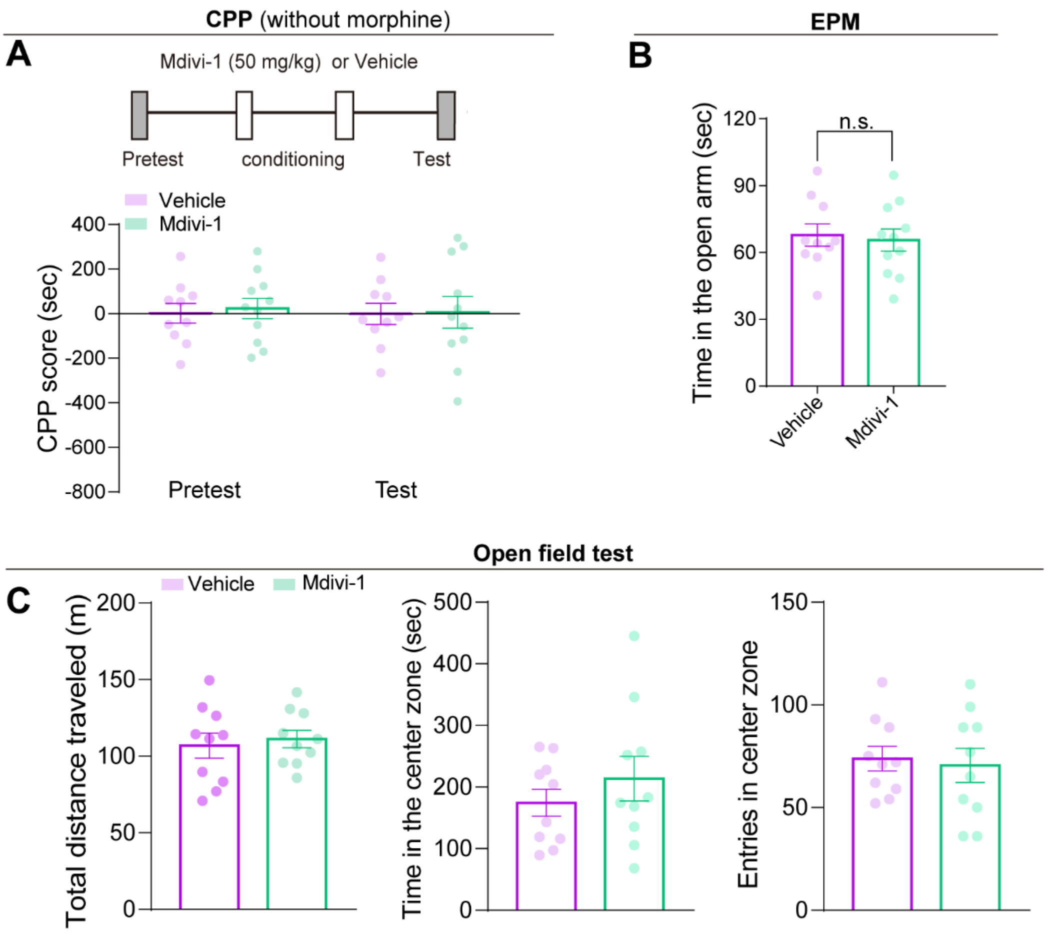

Figure S12. The effect of Mdivi-1 on the behavioral performance of morphine naive mice without chronic morphine administration. Related to Figure 11.

(**A**) Quantification of the conditioned place preference (CPP) scores of the mice from the Mdivi-1 and vehicle group. Mdivi-1 or vehicle was injected 45 min before each conditioning session. 10 mice/group, two-way RM ANOVA by Bonferroni's test. (**B** and **C**) The effect of Mdivi-1 on anxiety (**B**) and locomotor activity (**C**) in the EPM and open field tests were analyzed in the mice from the vehicle and Mdivi-1 groups. 10 mice/group, Unpaired t-test. Data are presented as mean ± S.E.M; *n.s.* not significant.

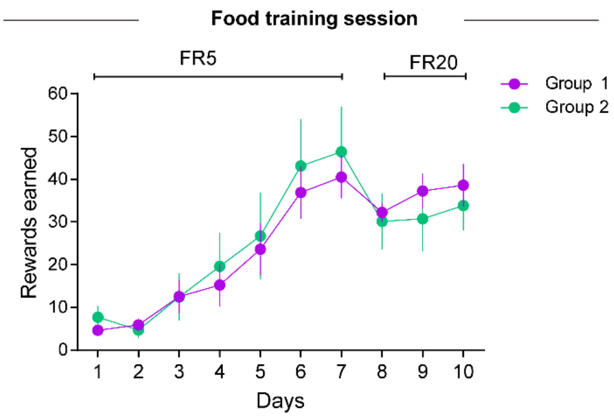

Figure S13. The learning curve of mice in food administration. Related to Figure 11.

Sucrose pellets number within 60 min of the mice divided into vehicle and Mdivi-1 groups in food administration. Two-way RM ANOVA, 7-8 mice/group. Data are presented as mean ± S.E.M.

Table S1. DNA Sequence of qRT-PCR Primer		
Gene	Primer	5‘-3’
gapdh	GAPDH-F	TGGCCTTCCGTGTTCCTAC
	GAPDH-R	GAGTTGCTGTTGAAGTCGCA
mfn1	mfn1-F	CCTACTGCTCCTTCTAACCCA
	mfn1-R	AGGGACGCCAATCCTGTGA
mfn2	mfn2-F	AAGGTTGAGGTGACAGCGTT
	mfn2-R	TCCACCTGTCCAAGCTTCTTC
opa1	opa1-F	TGGAAAATGGTTCGAGAGTCAG
	opa1-R	CATTCCGTCTCTAGGTTAAAGCG
fis1	Fis1-F	TGTCCAAGAGCACGCAATTTG
	Fis1-R	CCTCGCACATACTTTAGAGCCTT
dnm1l	dnm1l-F	CAGGAATTGTTACGGTTCCCTAA
	dnm1l-R	CCTGAATTAACTTGTCCCGTGA

Table S2. Key resources

REAGENT or RESOURCE	SOURCE	IDENTIFIER
Antibodies		
Rabbit anti-HA	Sigma-Aldrich	H6908; RRID:AB_260070
Mouse anti-TH	Millipore	MAB318; RRID:AB_2201528
Mouse anti-Nitrotyrosine	Santa Cruz	sc-32757; RRID:AB_628022
Rabbit anti-COXIV	Proteintech	Cat# 11242-1-AP; RRID:AB_2085278
Goat anti-mouse Alexa fluor cy3	Jackson ImmunoResearch	Cat# 115-005-003 RRID:AB_2338447
Goat anti-rabbit Alexa fluor cy3	Jackson ImmunoResearch	Cat# 111-165-144 RRID:AB_2338006
Goat anti-mouse Alexa fluor 488	Jackson ImmunoResearch	Cat# 115-545-166 RRID:AB_2338852
Goat anti-rabbit Alexa fluor 488	Jackson ImmunoResearch	Cat# 111-545-144 RRID:AB_2338052
In *situ* Probes		
EGFP	ACDbio	#400281-C2
Mfn1	ACDbio	#581881-C3
tdTomato	ACDbio	#317041-C2
Bacterial and virus strains		
pAAV-RAM-TTA-TRE-Flex-tdTomato	Addgene	RRID:Addgene_84468
pAAV-RAM-TTA-TRE-EGFP	Addgene	RRID:Addgene_84469
pAAV-hSyn-DIO-hM3D(Gq)-mCherry	Addgene	RRID:Addgene_44361
pAAV-Cre-GFP	Addgene	RRID:Addgene_68544
pAAV-hSyn-hM3Dq-mCherry	Addgene	RRID:Addgene_50474
pAAV-EF1α-Flpo	Addgene	RRID:Addgene_55637
pAAV-Ef1α-fDIO-hChR2(H134R)-EYFP	Addgene	RRID:Addgene_55639
p AAV.rTH.PI.Cre.SV40	Addgene	RRID:Addgene_107788
pAAV-Ef1α-DIO-EGFP	Addgene	RRID:Addgene_27056
pMfn1-Myc	Addgene	RRID:Addgene_23212
pAAV-DIO-jGCaMP7b	BrainVTA Co., Ltd	N/A
AAV-RAM-TTA-TRE-Flpo	Jiang et al.[18]	N/A
AAV-RAM-TTA-TRE-Flex-hM3Dq-HA	This manuscript	N/A
AAV- EF1α-fDIO-TH-Cre	This manuscript	N/A
AAV-DIO-4mito-jGCaMP7b	This manuscript	N/A
AAV-RAM-TTA-TRE-Flex-mito-tdTomato	This manuscript	N/A
AAV-DIO-Mfn1-EGFP	This manuscript	N/A
AAV-DIO-ChR2-EYFP	Taitool Bioscience Co., Ltd.	S0199
AAV-DIO-EGFP	Taitool Bioscience Co., Ltd.	S0270
AAV-EF1α-FLEX-NBL10	Taitool Bioscience Co., Ltd.	S0823
AAV-DIO-jGCaMP7b	BrainVTA Co., Ltd	PT-1423
AAV-hsyn-mt-keima	WZ Biosciences Co., Ltd	CV100200103
Chemicals, peptides, and recombinant proteins		
Morphine	Shenyang No.1 Pharmaceutical Co., Ltd	CFDA Approval# H21022436
Kaempferol	MedChemExpress	HY-14590; CAS: 520-18-3
Doxycycline (Dox)	MedChemExpress	HY-N0565; CAS: 564-25-0
Corn oil	Thermo scientific	405435000; CAS: 8001-30-7

Clozapine N-oxide (CNO)	Sigma-Aldrich	C0832; CAS: 34233-69-7
Mdivi-1	Sigma-Aldrich	M0199; CAS: 338967-87-6
L-Glutamic acid monosodium salt hydrate	Sigma-Aldrich	G1626; CAS: 142-47-2
L-(-)-Malic acid	Sigma-Aldrich	M1000; CAS: 636-61-3
Sodium succinate dibasic hexahydrate	Sigma-Aldrich	S2378; CAS: 6106-21-4
Malonic acid	Sigma-Aldrich	M1296; CAS: 141-82-2
Carbonyl cyanide 3-chlorophenylhydrazone (CCCP)	Sigma-Aldrich	C2759; CAS: 555-60-2
Calcium carbonate	Sigma-Aldrich	C4830; CAS: 471-34-1
Taurine	Sigma-Aldrich	T0625; CAS: 107-35-7
Phosphocreatine disodium salt hydrate	Sigma-Aldrich	P7936; CAS: 19333-65-4
MES hydrate	Sigma-Aldrich	M8250; CAS: 1266615-59-1
Adenosine 5′-diphosphate monopotassium salt dihydrate	Sigma-Aldrich	A5285; CAS: 72696-48-1
Cytochrome c from equine heart	Sigma-Aldrich	C7752; CAS: 9007-43-6
Oligomycin A	CSNpharm	CSN15998; CAS: 1397-94-0
Rotenone	CSNpharm	CSN11866; CAS: 83-79-4
AP-5	Tocris	Cat# 0106; CAS: 79055-68-8
Critical commercial assays		
RNAscope® 2.5 Universal Pretreatment Reagents	ACDbio	ACD: 322380
RNAscope® Multiplex Fluorescent Detection Kit v2	ACDbio	ACD: 323110
Experimental models: Organisms/strains		
Mouse: C57BL/6J	Silaike Experiment Animal Co., Ltd. (Shanghai)	JAX: 000664
Mouse: TH-Cre	The Jackson Laboratory	JAX stock # 008601
Software and algorithms		
Patchmaster	HEKA Elektronik	RRID:SCR_000034
Clampfit 10.3	Molecular Devices	RRID:SCR_011323
Nikon-NIS-Elements software	Nikon Corporation	N/A
MATLAB	MathWorks	RRID:SCR_001622
Activity Monitor software	MED Associates	RRID:SCR_014296
Image-Pro Plus 6.0	Media Cybernetics, Inc	RRID:SCR_016879
SPSS	IBM	RRID:SCR_002865
Prism	Graphpad Software	RRID:SCR_002798
Noldus	Noldus Information Tech	RRID:SCR_004074
